# Population genomic analysis of *Aegilops tauschii* identifies targets for bread wheat improvement

**DOI:** 10.1038/s41587-021-01058-4

**Published:** 2021-11-01

**Authors:** Kumar Gaurav, Sanu Arora, Paula Silva, Javier Sánchez-Martín, Richard Horsnell, Liangliang Gao, Gurcharn S. Brar, Victoria Widrig, W. John Raupp, Narinder Singh, Shuangye Wu, Sandip M. Kale, Catherine Chinoy, Paul Nicholson, Jesús Quiroz-Chávez, James Simmonds, Sadiye Hayta, Mark A. Smedley, Wendy Harwood, Suzannah Pearce, David Gilbert, Ngonidzashe Kangara, Catherine Gardener, Macarena Forner-Martínez, Jiaqian Liu, Guotai Yu, Scott A. Boden, Attilio Pascucci, Sreya Ghosh, Amber N. Hafeez, Tom O’Hara, Joshua Waites, Jitender Cheema, Burkhard Steuernagel, Mehran Patpour, Annemarie Fejer Justesen, Shuyu Liu, Jackie C. Rudd, Raz Avni, Amir Sharon, Barbara Steiner, Rizky Pasthika Kirana, Hermann Buerstmayr, Ali A. Mehrabi, Firuza Y. Nasyrova, Noam Chayut, Oadi Matny, Brian J. Steffenson, Nitika Sandhu, Parveen Chhuneja, Evans Lagudah, Ahmed F. Elkot, Simon Tyrrell, Xingdong Bian, Robert P. Davey, Martin Simonsen, Leif Schauser, Vijay K. Tiwari, H. Randy Kutcher, Pierre Hucl, Aili Li, Deng-Cai Liu, Long Mao, Steven Xu, Gina Brown-Guedira, Justin Faris, Jan Dvorak, Ming-Cheng Luo, Ksenia Krasileva, Thomas Lux, Susanne Artmeier, Klaus F. X. Mayer, Cristobal Uauy, Martin Mascher, Alison R. Bentley, Beat Keller, Jesse Poland, Brande B. H. Wulff

**Affiliations:** 1 John Innes Centre, Norwich Research Park, Norwich, UK; 2 Department of Plant Pathology and Wheat Genetics Resource Center, Kansas State University, Manhattan, KS USA; 3 Programa Nacional de Cultivos de Secano, Instituto Nacional de Investigación Agropecuaria (INIA), Estación Experimental La Estanzuela, Colonia, Uruguay; 4 Department of Plant and Microbial Biology, University of Zurich, Zurich, Switzerland; 5 The John Bingham Laboratory, NIAB, Cambridge, UK; 6 Crop Development Centre, Department of Plant Sciences, University of Saskatchewan, Saskatoon, Saskatchewan Canada; 7 Faculty of Land and Food Systems, The University of British Columbia, Vancouver, British Columbia Canada; 8 Leibniz-Institute of Plant Genetics and Crop Plant Research (IPK) Gatersleben, Seeland, Germany; 9 National Key Laboratory of Crop Genetics and Germplasm Enhancement, Cytogenetics Institute, Nanjing Agricultural University/JCIC-MCP, Nanjing, China; 10 School of Agriculture, Food and Wine, University of Adelaide, Glen Osmond, South Australia Australia; 11 Department of Agricultural and Food Sciences, Alma Mater Studiorum, University of Bologna, Bologna, Italy; 12 Department of Agroecology, Global Rust Reference Center, Aarhus University, Slagelse, Denmark; 13 Texas A&M AgriLife Research, Amarillo, TX USA; 14 Institute for Cereal Crops Improvement, School of Plant Sciences and Food Security, Tel Aviv University, Tel Aviv, Israel; 15 Department of Agrobiotechnology (IFA-Tulln), Institute of Biotechnology in Plant Production, University of Natural Resources and Life Sciences, Vienna, Austria; 16 Laboratory of Plant Breeding, Department of Agronomy, Faculty of Agriculture, Universitas Gadjah Mada, Yogyakarta, Indonesia; 17 Department of Agronomy and Plant Breeding, Ilam University, Ilam, Iran; 18 Institute of Botany, Plant Physiology and Genetics, Tajik National Academy of Sciences, Dushanbe, Tajikistan; 19 Germplasm Resources Unit, John Innes Centre, Norwich Research Park, Norwich, UK; 20 Department of Plant Pathology, University of Minnesota, Saint Paul, MN USA; 21 School of Agricultural Biotechnology, Punjab Agricultural University, Ludhiana, India; 22 Commonwealth Scientific and Industrial Research Organization (CSIRO), Agriculture and Food, Canberra, Australian Capital Territory Australia; 23 Wheat Research Department, Field Crops Research Institute, Agricultural Research Center, Giza, Egypt; 24 Earlham Institute, Norwich Research Park, Norwich, UK; 25 QIAGEN Aarhus A/S, Aarhus, Denmark; 26 Department of Plant Science and Landscape Architecture, University of Maryland, College Park, MD USA; 27 Institute of Crop Science, Chinese Academy of Agricultural Sciences, Beijing, China; 28 Triticeae Research Institute, Sichuan Agricultural University, Chengdu, China; 29 USDA-ARS Cereal Crops Research Unit, Edward T. Schafer Agricultural Research Center, Fargo, ND USA; 30 USDA-ARS, Plant Science Research Unit, Raleigh, NC USA; 31 Department of Plant Sciences, University of California, Davis, CA USA; 32 Department of Plant and Microbial Biology, University of California, Berkeley, CA USA; 33 Plant Genome and Systems Biology, Helmholtz Center Munich, Neuherberg, Germany; 34 Faculty of Life Sciences, Technical University Munich, Weihenstephan, Germany; 35 German Centre for Integrative Biodiversity Research (iDiv) Halle-Jena-Leipzig, Leipzig, Germany; 36 Present Address: Bayer R&D Services LLC, Kansas City, MO USA; 37 Present Address: Center for Desert Agriculture, Biological and Environmental Science and Engineering Division (BESE), King Abdullah University of Science and Technology (KAUST), Thuwal, Saudi Arabia; 38 Present Address: International Maize and Wheat Improvement Center (CIMMYT), Texcoco, Mexico

**Keywords:** Plant domestication, Genome informatics, Plant breeding, Plant immunity, Genome-wide association studies

## Abstract

*Aegilops tauschii*, the diploid wild progenitor of the D subgenome of bread wheat, is a reservoir of genetic diversity for improving bread wheat performance and environmental resilience. Here we sequenced 242 *Ae. tauschii* accessions and compared them to the wheat D subgenome to characterize genomic diversity. We found that a rare lineage of *Ae. tauschii* geographically restricted to present-day Georgia contributed to the wheat D subgenome in the independent hybridizations that gave rise to modern bread wheat. Through *k*-mer-based association mapping, we identified discrete genomic regions with candidate genes for disease and pest resistance and demonstrated their functional transfer into wheat by transgenesis and wide crossing, including the generation of a library of hexaploids incorporating diverse *Ae. tauschii* genomes. Exploiting the genomic diversity of the *Ae. tauschii* ancestral diploid genome permits rapid trait discovery and functional genetic validation in a hexaploid background amenable to breeding.

## Abstract (web summary)

Exploring the diversity of the wild progenitor of the wheat D subgenome allows for rapid trait discovery.

## Main

The success of bread wheat (*Triticum aestivum*) as a major worldwide crop is underpinned by its adaptability to diverse environments, high grain yield and nutritional content^1^. With the combined challenge of population expansion and hotter, less favorable climates, wheat yields must be sustainably increased to ensure global food security. The rich reservoir of genetic diversity amongst the wild relatives of wheat provides a means to improve productivity^1,2^. Maximizing the genetic potential of wheat requires a deep understanding of the structure and function of its genome, including its relationship with its wild progenitor species.

The evolution of bread wheat from its wild relatives is typically depicted as two sequential interspecific hybridization and genome duplication events leading to the genesis of the allohexaploid bread wheat genome^2,3^. The first hybridization between *T*. *urartu* (AA) and a presumed extinct diploid (BB) species formed tetraploid emmer wheat, *T*. *turgidum* (AABB), ~0.5 million years ago^4^. The gradual process of domestication of *T. turgidum* started with its cultivation in the Fertile Crescent some 10,000 years ago^5^. Subsequent hybridization with *Ae. tauschii* (DD) formed the hexaploid *T*. *aestivum* (AABBDD)^6^. Whereas ancient gene flow incorporated the majority of the AABB genome diversity into hexaploid wheat, only a small fraction of the D genome diversity was captured^7^. Indeed, hybridization between *T. turgidum* and *Ae. tauschii* was thought to be restricted to a subpopulation of *Ae. tauschii* from the shores of the Caspian Sea in present-day Iran^8^. Despite sampling limited diversity, this genomic innovation created a plant more widely adapted to a broad range of environments and with end-use qualities not found in its progenitors^1^.

The low genetic diversity of the bread wheat D subgenome has long motivated breeders to recruit diversity from *Ae. tauschii*. The most common route involves hybridization between tetraploid wheat and *Ae. tauschii* followed by chromosome doubling to create synthetic hexaploids^9^. Alternatively, direct hybridization between hexaploid wheat and *Ae. tauschii* is possible. This approach usually requires embryo rescue but has the advantage that it does not disrupt desirable allele combinations in the bread wheat A and B subgenomes^10,11^. Notwithstanding, the products of all these wide crosses require backcrossing to domesticated cultivars to remove unwanted agronomic traits from the wild progenitor and restore optimal end-use qualities. The boost to genetic diversity and resilience therefore comes at a cost to the breeder^9^. However, if haplotypes underlying useful traits could be directly identified in *Ae. tauschii*, this would mitigate a critical limitation in breeding wheat with *Ae. tauschii*; such haplotypes can be tagged with molecular markers for accelerated delivery into domesticated wheat by combining marker-assisted selection^12^ with rapid generation advancement^13^. Furthermore, a gene-level understanding would permit next-generation breeding by gene editing and transformation.

In this study, we performed whole-genome shotgun short-read sequencing on a diverse panel of 242 *Ae. tauschii* accessions. We discovered that an uncharacterized *Ae. tauschii* lineage contributed to the initial gene flow into domesticated wheat, thus broadening our understanding of the evolution of bread wheat. To facilitate the discovery of useful genetic variation from *Ae. tauschii*, we established a *k*-mer-based association mapping pipeline and demonstrated the mobilization of the untapped diversity from *Ae. tauschii* into wheat through the use of synthetic wheats and genetic transformation for biotic stress resistance genes.

## Results

### Multiple hybridizations shaped the bread wheat D subgenome

**Fig. 1:**
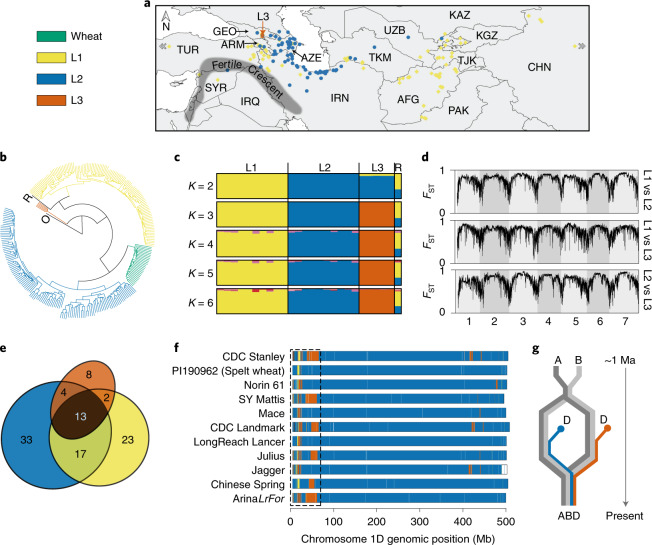
Characterization of a third lineage of *Ae. tauschii* and its contribution to the wheat D subgenome. The color code for all panels is shown for wheat and *Ae. tauschii* lineages (L1, L2, L3) in the top left corner. **a**, Distribution of the 242 *Ae. tauschii* samples used in this study. The five L3 accessions are indicated by an orange vertical arrow. Country abbreviations are provided in Extended Data Fig. 1a. **b**, Phylogeny showing the D subgenome of 28 wheat landraces in relation to *Ae. tauschii*, a tetraploid (AABB genome) outgroup (O) and an *Ae. tauschii* RIL (labeled R) derived from L1 and L2. **c**, STRUCTURE analysis of the randomly selected ten accessions from each of L1 and L2 along with the five accessions of L3 and the RIL. *K* denotes the number of subpopulations considered. **d**, Genome-wide fixation index (*F*_ST_) estimates of the *Ae. tauschii* lineages. **e**, Venn diagram showing the percentage of lineage-specific and shared *k*-mers between the lineages. **f**,**g**, Chromosome 1D of wheat cultivars/accessions colored according to their *Ae. tauschii* lineage-specific origin (**f**). The pattern of lineage-specific contribution to the wheat D subgenome, highlighted for one region by a dashed rectangle, suggests that at least two polyploidization events with distinct *Ae. tauschii* lineages, as shown in **g**, followed by intraspecific crossing gave rise to extant hexaploid bread wheat. Ma, million years ago.

**Extended Data Fig. 1:**
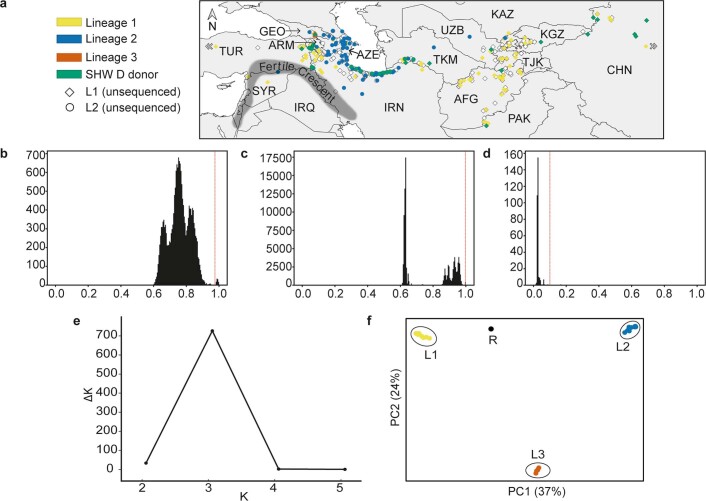
Configuration and genetic structure of the *Aegilops tauschii* diversity panel used in this study. **a**, Geographical distribution of 242 *Ae. tauschii* accessions. Filled squares and circles represent accessions sequenced as part of this study, while accessions represented by unfilled squares and circles were not sequenced. Accessions highlighted in green were used as D genome donors to generate synthetic hexaploid wheat (SHW) lines. Three accessions outside of the map, one from Turkey and two from China, are indicated by white arrow heads. AFG, Afghanistan; ARM, Armenia; AZE, Azerbaijan; CHN, China; GEO, Georgia; IRN, Iran; IRQ, Iraq; KAZ, Kazakhstan; KGZ, Kyrgyzstan; PAK, Pakistan; SYR, Syria; TJK, Tajikistan; TUR, Turkey; TKM, Turkmenistan; UZB, Uzbekistan. The Fertile Crescent follows the shaded area in Fig. 1 of Harlan and Zohary (1966) and is bound by the Mediterranean in the west, by chains of large and high mountain ranges in the north and east (the Amanos in northwestern Syria, the Taurus in southern Turkey, Ararat in north-eastern Turkey and the Zagros in western Iran), and in the south by the Syrio-Arabian desert, with its western extension (for example, Paran desert) in the Sinai Peninsula. **b**, Identification of non-redundant *Ae. tauschii* accessions using KASP markers on 195 accessions and: **c**, 100,000 random SNPs obtained from whole genome shotgun sequencing of 306 accessions. The vertical red line in both histogram similarity plots indicates the redundancy cut-off at which the peak of the high similarity values is clearly separated from the rest. **d**, Identification of *Ae. tauschii* accessions with minimal residual heterogeneity. The histogram of heterozygosity scores was generated using all the bi-allelic SNPs obtained from whole genome shotgun sequencing of 305 accessions (excluding TOWWC0193). The vertical red line indicates the cut-off at which the cluster of the low heterozygosity values is clearly separated. **e**, ΔK plot for a STRUCTURE run with 10 randomly selected accessions each of L1 and L2 along with the five accessions of the putative L3 and the control L1-L2 RIL. **f**, Principal Component Analysis with the same set of accessions as used in panel a. The recombinant inbred control line is indicated by R.

We identified a set of 242 non-redundant *Ae. tauschii* accessions with minor residual heterogeneity after short-read sequencing of 306 accessions covering the geographical range spanned by diverse *Ae. tauschii* collections (Fig. 1a, Extended Data Fig. 1a–d, Supplementary Tables 1–5 and Supplementary Note). To capture the genetic diversity of the *Ae. tauschii* species complex, we generated a *k*-mer matrix specifying the presence and absence of a comprehensive set of 51-mer variants in the sequenced accessions and a single-nucleotide polymorphism (SNP) matrix relative to the AL8/78 reference genome^14^.

*Ae. tauschii* is generally categorized into two lineages, lineage 1 (L1) and lineage 2 (L2)^15,16^, with L2 considered the major contributor to the wheat D subgenome^8^. To better understand the relationship between *Ae. tauschii* and wheat, we randomly selected 100,000 *k*-mers and checked their presence in the short-read sequences of 28 hexaploid wheat landraces^17^. We used a tetraploid wheat accession as an outgroup in the phylogenetic analysis and included a recent *Ae. tauschii* L1–L2 recombinant inbred line (RIL)^15^ as a control in our population structure analysis. We generated a phylogeny based on the presence/absence of these *k*-mers and found it to be consistent with earlier phylogenies generated using molecular markers in that *Ae. tauschii* L1 and L2 formed two major clades, whereas the wheat D subgenome formed a discrete and narrow clade most closely related to L2 (Fig. 1b)^8,15,16^. This supports the L2 origin of the wheat D subgenome and its limited genetic diversity relative to *Ae. tauschii*. A group of five accessions formed a distinct clade separate from L1 and L2, as previously observed^15,16^, which seems to be a basal lineage based on the split from the outgroup. Matsuoka et al. hypothesized that this group could be a separate lineage^18^, whereas Singh et al. hypothesized that it could have arisen from interlineage hybridization followed by isolated evolution^15^. To resolve this question, we conducted Bayesian clustering analysis using STRUCTURE^19^. Because this algorithm does not reliably recover the correct population structure when sampling is uneven^20^, we randomly selected ten accessions from L1 and from L2 for this analysis along with the five accessions of the putative lineage 3 (L3) and the control L1–L2 RIL (Supplementary Table 6). Performing STRUCTURE analysis with the number of subpopulations, *K* = 2 showed the putative L3 accessions as an admixture of L1 and L2, similar to the L1–L2 RIL; but with *K* = 3, these accessions were assigned to a distinct lineage (Fig. 1c). Further increasing the value of *K* did not reveal any discernible substructure. This interpretation was supported by the Δ*K* curve, which showed a clear peak at *K* = 3 (Extended Data Fig. 1e). Principal-component analysis (PCA) also separated the population into three clusters corresponding to L1, L2 and L3 (Extended Data Fig. 1f). Computing the genome-wide pairwise fixation index (*F*_ST_) between the three lineages using SNPs in a sliding window of 1 megabases (Mb) with a step size of 100 kilobases (kb) indicated a high level of population differentiation across the genome, with values near 1.0 in the centromeric regions and around 0.3–0.5 near the telomeric ends (Fig. 1d). These observations demonstrate the existence of a differentiated third lineage within *Ae. tauschii*.

**Extended Data Fig. 2:**
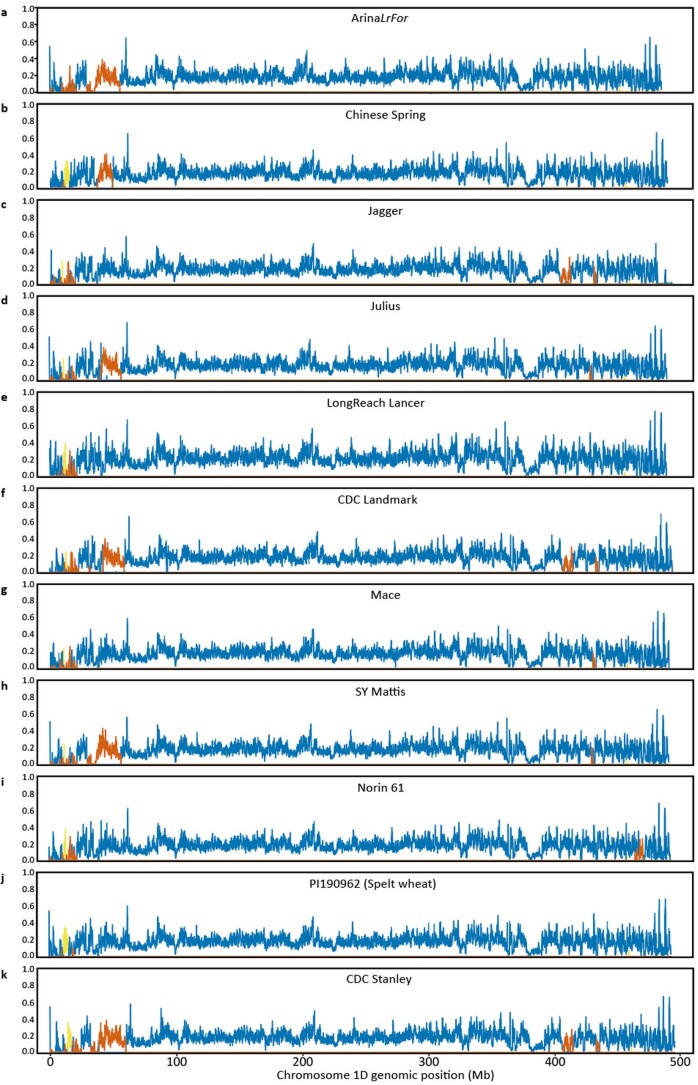
Fraction of lineage-specific *k*-mers in non-overlapping 100 kb windows of Chromosome 1D for the 11 wheat genome assemblies. For the nine modern cultivars^21^, only those *k*-mers were considered which were also present in the short-read sequences of 28 hexaploid wheat landraces^17^. Chromosomes are colored according to their *Ae. tauschii* lineage-specific origin as displayed in Fig. 1.

**Extended Data Fig. 3:**
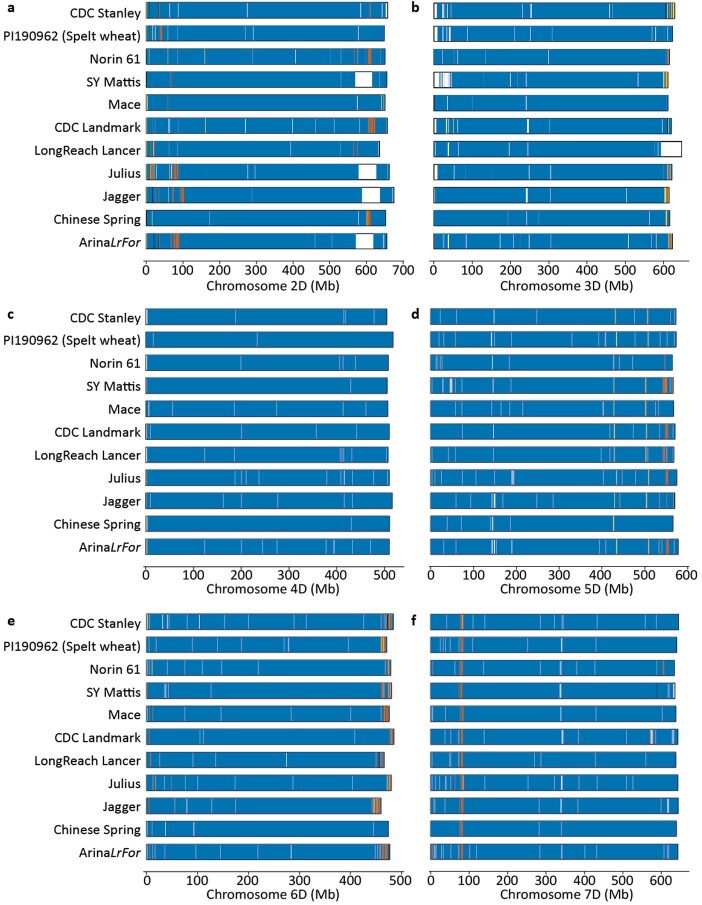
Lineage-specific origin of extant wheat D-subgenomes. Chromosomes 2D-7D of 11 wheat cultivars colored according to their *Ae. tauschii* lineage-specific origin as in Fig. 1f.

Consistent with the above population structure, we found that 64% of the *Ae. tauschii k*-mer space, obtained by summing up the percentages in the non-overlapping sections of the Venn diagram (Fig. 1e), is lineage specific. We used the lineage-specific *k*-mers to understand the origin of the wheat D subgenome by representing the D subgenomes of the available chromosome-scale wheat assemblies^21^ as 100-kb segments and assigning them to the *Ae. tauschii* lineage predominantly contributing lineage-specific *k*-mers to that segment (Extended Data Fig. 2). To account for recent alien introgressions in modern cultivars due to breeding, only those *k*-mers that were also present in the 28 hexaploid wheat landraces^17^ were used. The differential presence of L2 and L3 segments at multiple independent regions in these wheat lines (shown for chromosome 1D in Fig. 1f and chromosomes 2D–7D in Extended Data Fig. 3) suggests that at least two hybridization events gave rise to the extant wheat D subgenome (Fig. 1g) and that one of the D genome donors was of predominantly L2 origin, while the other was of predominantly L3 origin. The total L3 contribution across all the seven chromosomes ranges from 0.5% for Spelt, *T. aestivum* spp. *spelta*, to 1.9% for *T. aestivum* ssp. *aestivum ArinaLrFor*, with an average of 1.1% for all the 11 reference genomes (Extended Data Fig. 3).

### Discovery of *Ae. tauschii* trait–genotype correlations

**Fig. 2:**
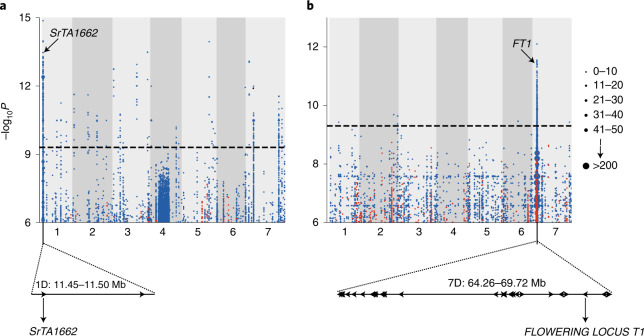
Genetic identification of candidate genes for stem rust resistance and flowering time by *k*-mer-based association mapping. **a**, *k*-mers significantly associated with resistance to *Puccinia graminis* f. sp. *tritici* race QTHJC mapped to scaffolds of a de novo assembly of *Ae. tauschii* accession TOWWC0112 anchored to chromosomes 1 to 7 of the D subgenome of Chinese Spring^51^. Points on the *y* axis show *k*-mers significantly associated with resistance (blue) and susceptibility (red). **b**, *k*-mers significantly associated with flowering time mapped to *Ae. tauschii* reference genome AL8/78 with early (red) or late (blue) flowering time association relative to the population mean across the diversity panel. Candidate genes for both phenotypes are highlighted. Point size is proportional to the number of *k*-mers (see inset). The association score is defined as the –log_10_ of the *P* value obtained using the likelihood ratio test for nested models. The threshold of significant association scores is adjusted for multiple comparisons using the Bonferroni method.

**Extended Data Fig. 4:**
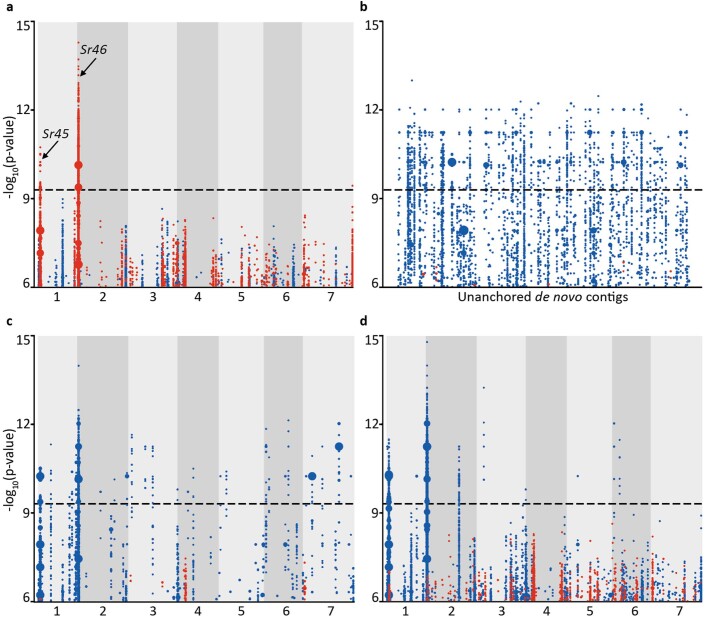
Optimization of *k*-mer GWAS with the positive controls *Sr45* and *Sr46*. Blue/red dots on the y-axis represent one or more *k*-mers significantly associated with resistance/susceptibility, respectively, to *Puccinia graminis* f. sp. *tritici* isolate 04KEN156/04 (race TTKSK) across the diversity panel. Definition of association score, threshold, and dot size (which is proportional to the number of *k*-mers having the specific value on the y-axis), is as in Fig. 2. **a**, Significantly associated *k*-mers mapped to AL8/78 which is susceptible to TTKSK. The peaks marked *Sr45* and *Sr46* contain the non-functional (not providing resistance to TTKSK) alleles of *Sr45* and *Sr46*. The x-axis represents the seven chromosomes of *Ae. tauschii* reference accession, AL8/78. Each dot column represents a 10 kb interval. **b**, Significantly associated *k*-mers mapped to the unordered *de novo* assembly of TOWWC0112 (N50 1.1 kb), an *Ae. tauschii* accession resistant to TTKSK. Each dot-column on the x-axis represents an unordered contig from the de novo assembly. **c**, Significantly associated *k*-mers mapped to the same assembly of TOWWC0112 as in (b), but now each contig has been ordered by anchoring to the reference genome of AL8/78 (x-axis). **d**, Association mapping with an improved TOWWC0112 assembly (N50 196 kb) anchored to the AL8/78 reference genome (x-axis).

**Extended Data Fig. 5:**
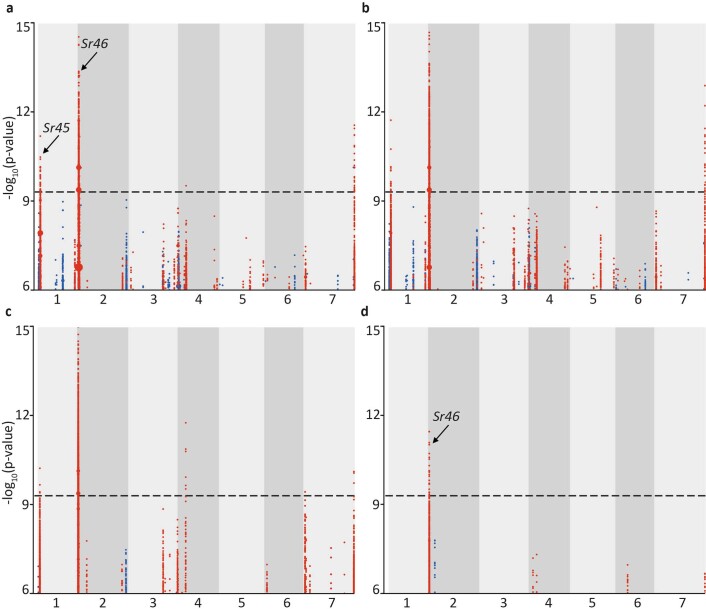
Impact of sequencing coverage on the power to detect the positive controls, *Sr45* and *Sr46*. Sequencing coverage was artificially reduced by sub-sampling the original 10-fold coverage sequencing reads and mapping associated *k*-mers to AL8/78. Definition of association score, threshold, and dot size is as in Fig. 2. **a**, Plot obtained with 7.5-fold coverage (compare with 10-fold coverage in Extended Data Fig. 7a). **b**, Plot obtained with 5-fold coverage. **c**, Plot obtained with 3-fold coverage. **d**, Plot obtained with 1-fold coverage.

**Extended Data Fig. 6:**
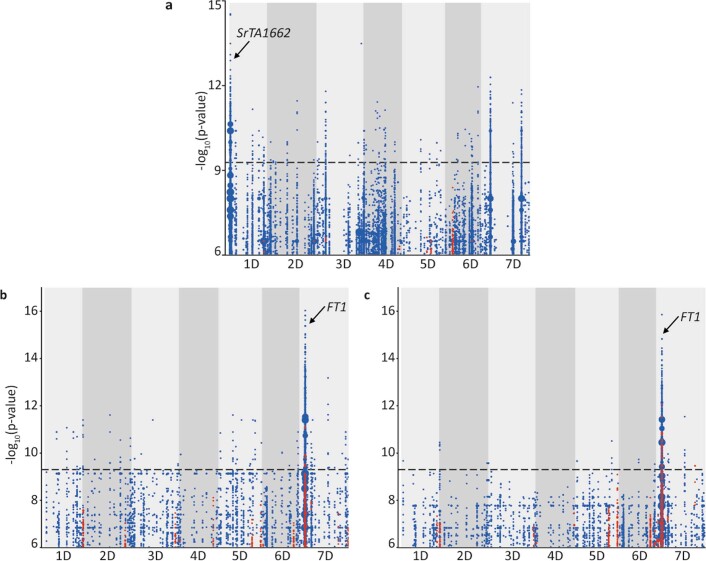
*k*-mers significantly associated with *FLOWERING LOCUS T1* and *SrTA1662* identified by GWAS. Definition of association score, threshold, and dot size is as in Fig. 2. **a**, Resistance to *Puccinia graminis* f. sp. *tritici* isolate UK-01 maps to the *SrTA1662* locus. The peak indicated by the arrow contains the region delimited by the *SrTA1662* LD block obtained with *P. graminis* f. sp. *tritici* race QTHJC. **b**, Biological replicate 2 and **c**, biological replicate 3 for flowering time identify *FLOWERING LOCUS T1*. The associated *k*-mers were mapped to the *Aegilops tauschii* AL8/78 reference genome where they define a peak similar to that in Fig. 2b.

Identification of genes or haplotypes in *Ae. tauschii* underpinning useful variation would permit accelerated wheat improvement through wide crossing and marker-assisted selection or biotechnological approaches to introduce them into wheat. To identify this variation, we adapted our *k*-mer-based association mapping pipeline, previously developed for resistance gene families obtained using sequence capture^22^, to whole-genome shotgun data (Extended Data Fig. 4a). The significantly associated *k*-mers were not just directly mapped to the *Ae. tauschii* AL8/78 reference genome but were also mapped to the de novo assembly of a relevant accession, which was anchored to the reference genome. In theory, using a set of de novo assemblies (either reference or anchored to a reference) covering the species diversity in this manner would enable us to determine the genomic context of all the significant *k*-mers. To demonstrate the advantage of this approach, we generated a de novo assembly of accession TOWWC0112 (N50 = 196 kb; Supplementary Table 7), which carries two cloned stem rust resistance genes that could be used as controls, and then anchored this assembly to a reference genome^14^. This enabled identification of the *cis*-associated *k*-mers rather than those linked in repulsion to the corresponding region in the reference genome (Extended Data Fig. 4c,d). Note the improvement in the association signal with the improvement in the quality of de novo assembly; when the quality is poor (Extended Data Fig. 4c), some of the short scaffolds with the significant *k*-mers are anchored outside the true locus, but with the improved de novo assembly (Extended Data Fig. 4d), most of the scaffolds with the significant *k*-mers tend to concentrate around the true locus. We also determined that the sequencing coverage could be reduced from tenfold to fivefold with no appreciable loss of signal from the two control genes (Extended Data Fig. 5). To test our method further, we performed association mapping for resistance to additional stem rust isolates and flowering time. For stem rust, we identified a peak within the genetic linkage group of *SrTA1662* (ref. ^23^) (Fig. 2a, Extended Data Fig. 6a and Supplementary Table 8). Annotation of the associated 50-kb linkage disequilibrium (LD) block revealed two genes, of which one encoded the nucleotide-binding and leucine-rich repeat (NLR) gene previously identified in our sequence capture association pipeline^22^ (Fig. 2a, Supplementary Tables 9 and 10 and Supplementary Note). We also recorded flowering time and found that it mapped to a broad peak of 5.46 Mb on chromosome arm 7DS containing 35 genes, including *FLOWERING LOCUS T1* (Fig. 2b, Extended Data Fig. 6b,c and Supplementary Tables 8 and 10), a well-known regulator of flowering time in dicots and monocots, including wheat^24–26^.

**Fig. 3:**
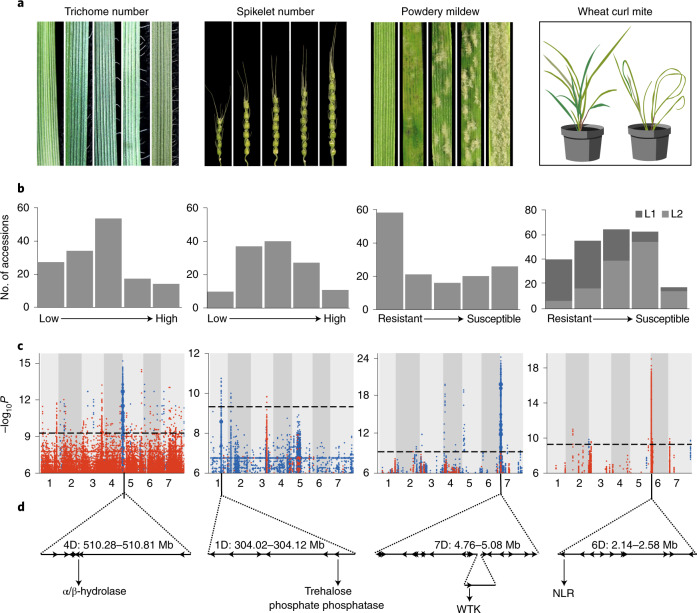
Genome-wide association mapping in *Ae. tauschii* for morphology, disease and pest resistance traits. **a**, Representation of the scale of phenotypic variation observed. **b**, Frequency distribution of the different phenotypic scales corresponding to **a**. L1 and L2 are shown in dark and light gray, respectively. **c**, *k*-mer–based association mapping to a de novo assembly of accession TOWWC0112 anchored to the AL8/78 reference genome (trichome number, spikelet number) or accession TOWWC0106 anchored to AL8/78 (response to powdery mildew) or directly mapped to AL8/78 (response to wheat curl mite). *k*-mer color coding, association score, threshold and dot size are as in Fig. 2. **d**, Identification of genes under the peak in the GWAS plot with promising candidate(s) indicated. The *WTK* gene resides within a 60-kb insertion relative to the AL8/78 reference genome.

**Extended Data Fig. 7:**
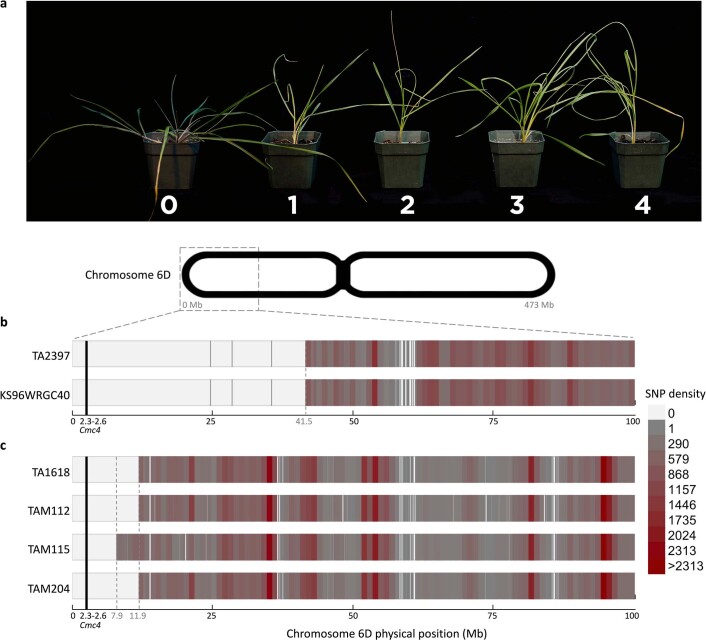
Wheat curl mite (WCM) symptoms in *Aegilops tauschii* and introgression of WCM resistance into wheat. **a**, Phenotype scale used to characterize *Ae. tauschii* response to WCM infestation. Symptoms used were leaf trapping and leaf curliness. The visual scale ranged from 0 to 4, with 0 equivalent to no symptoms and 1 to 4 denoting increasing levels of curliness or trapped leaves indicative of susceptibility. **b**, Delineation of *Ae. tauschii* Lineage 1 accession TA2397 carrying wheat curl mite resistance introgressed into wheat line KS96WGRC40. The retained polymorphic markers were obtained by pairwise comparisons of the *Ae. tauschii* donor with the corresponding wheat line. KS96WGRC40 is the original line where *Cmc4* was mapped. **c**, The donor of resistance in wheat line TAM 112 is the Lineage 2 accession TA1618. Wheat lines TAM 115 and TAM 204 are both resistant through TAM 112. The black vertical line indicates the *Cmc4* position. The three grey dashed vertical lines denote the size of the introgressed fragments, 7.9 Mb, 11.9 Mb, and 41.5 Mb, in the wheat lines TAM 115, TAM 112 and TAM 204, and KS96WGRC40, respectively. SNP density is based on number of SNPs within 1 Mb bins.

**Extended Data Fig. 8:**
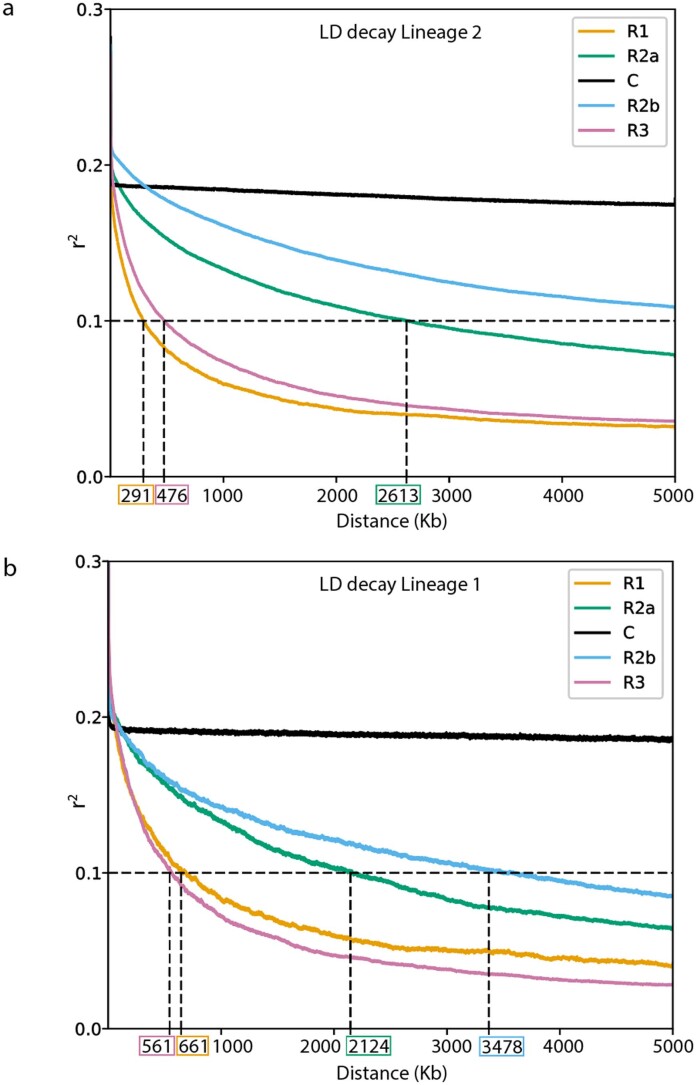
Genome-wide decay of linkage disequilibrium (LD) in *Aegilops tauschii*. Genomic regions (R1, R2a, C, R2b, R3) in L2 (top) and L1 (bottom) were determined based on the distribution of the recombination rate in *T. aestivum* cv. Chinese Spring. The distance at which r^2^ for a region drops below 0.1 is highlighted.

We next screened the *Ae. tauschii* panel for leaf trichomes (a biotic and abiotic resilience trait^27,28^), spikelet number per spike (a yield component), infection by *Blumeria graminis* f. sp. *tritici* (cause of powdery mildew) and resistance to the wheat curl mite *Aceria tosichella* (vector of wheat streak mosaic virus)^29^ (Supplementary Table 8). All four phenotypes presented continuous variation in the panel (Fig. 3a,b and Extended Data Fig. 7a). Mean trichome number along the leaf margin mapped to a 530-kb LD block on chromosome arm 4DL (Fig. 3c,d and Supplementary Table 10) within a 12.5-cM region previously defined by biparental linkage mapping^30^. The 530-kb interval contains seven genes, including an α/β-hydrolase, a gene class with increased transcript abundance in developing trichomes of *Arabidopsis thaliana*^31^. The number of spikelets per spike was associated with a discrete 100-kb peak on chromosome arm 1DL containing six genes (Fig. 3c,d and Supplementary Table 10). One of these encodes a trehalose-6-phosphate phosphatase that is homologous to RAMOSA3 and TPP4, known to control inflorescence branch number in maize^32^, and SISTER OF RAMOSA3 that influences spikelet fertility in barley^33^. Powdery mildew resistance mapped to a 320-kb LD block on chromosome arm 7DS containing 19 genes in the resistant haplotype, including a ~60-kb insertion with respect to the reference genome AL8/78 (Fig. 3c,d and Supplementary Table 10). No NLR immune receptor-encoding gene was detected; however, the insertion contains a wheat-tandem kinase (WTK), a gene class previously reported to confer resistance to wheat stripe rust (*Yr15*)^34^, stem rust (*Rpg1* and *Sr60*)^35,36^ and powdery mildew (*Pm24*)^37^. Resistance to wheat curl mite mapped to a 440-kb LD block on chromosome arm 6DS within a region previously determined by biparental mapping^38–40^ (Fig. 3c,d, Supplementary Table 10 and Supplementary Note). The interval contained ten genes, including an NLR immune receptor, a gene class previously reported to confer arthropod resistance in melon and tomato^41^. These results highlight the ability of the panel, with its rapid LD decay (Extended Data Fig. 8) and *k*-mer-based association mapping combined with de novo genome assembly and annotation, to identify candidate genes, including those in insertions with respect to the reference genome, within discrete genomic regions for quantitative traits of agronomic value.

### L1 and L2 share regions of low genetic divergence

**Fig. 4:**
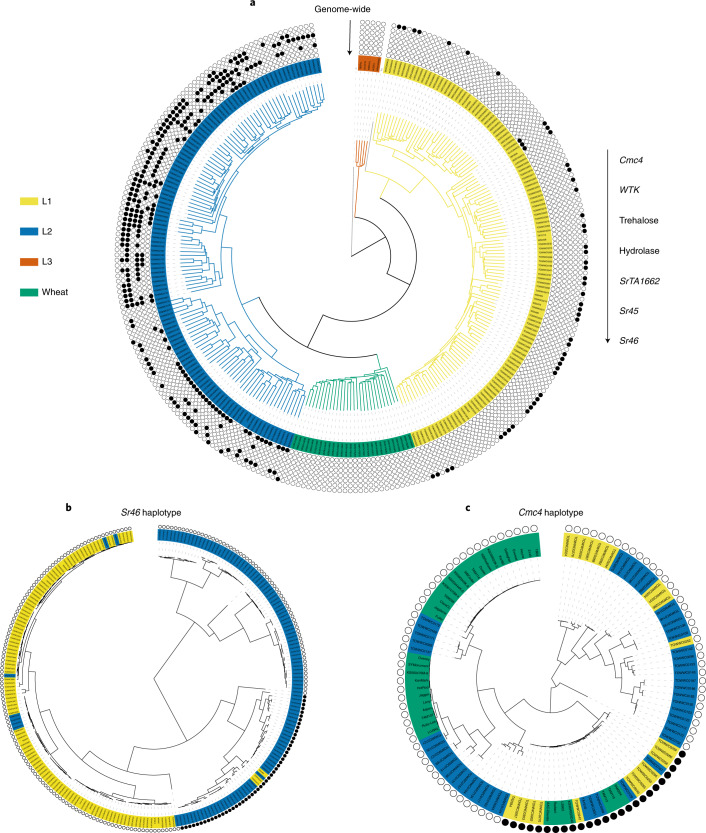
Comparison of genome-wide phylogeny with phylogenies of haplotypes surrounding specific genes. **a**, Genome-wide *k*-mer-based phylogeny of *Ae. tauschii* and hexaploid wheat landraces with designation of the presence of candidate and cloned genes/alleles for disease and pest resistance and morphological traits. The presence and absence of allele-specific polymorphisms is indicated by circles filled with black or white, respectively, for all but outgroup and RIL (gray edges). **b**, ﻿Phylogeny of *Ae. tauschii* L1 and L2 accessions based on SNPs restricted to the 200-kb region surrounding *Sr46*. **c**, Phylogeny based on SNPs of the 440-kb region in LD with *Cmc4*. Only the most resistant and susceptible *Ae. tauschii* accessions were included, along with resistant and susceptible modern elite wheat cultivars (different from the landraces shown in **a**).

We investigated the population-wide distribution of the candidate genes controlling disease resistance and morphology identified by association mapping (Figs. 2 and 3) across a genome-wide phylogeny of *Ae. tauschii* and a worldwide collection of 28 wheat landraces^17^. The absence of the alleles promoting disease resistance, more spikelets and higher trichome density in the wheat landraces for the new candidate genes suggest that they were not incorporated into the initial gene flow into wheat (Fig. 4a). We next examined the distribution of these alleles between the three lineages of *Ae. tauschii*. The *Cmc4* gene candidate for resistance to wheat curl mite was largely confined to L1, whereas the allele variants promoting higher trichome density, spikelet number and resistance to wheat stem rust and powdery mildew were largely confined to L2 (Fig. 4a). Exceptions included three occurrences of the *Sr46* gene in L1 and five occurrences of the candidate *Cmc4* gene in L2. To investigate whether this was due to a common genetic origin or convergent evolution, we generated phylogenies based on the SNPs within the respective 200-kb and 440-kb *Sr46* and *Cmc4* LD blocks. This showed that all functional haplotypes clustered together irrespective of genome-wide lineage assortment, indicative of a common genetic origin and not convergent evolution (Fig. 4b,c, Supplementary Table 11 and Supplementary Note).

### After domestication delivery of *Ae. tauschii* genes into wheat

**Fig. 5:**
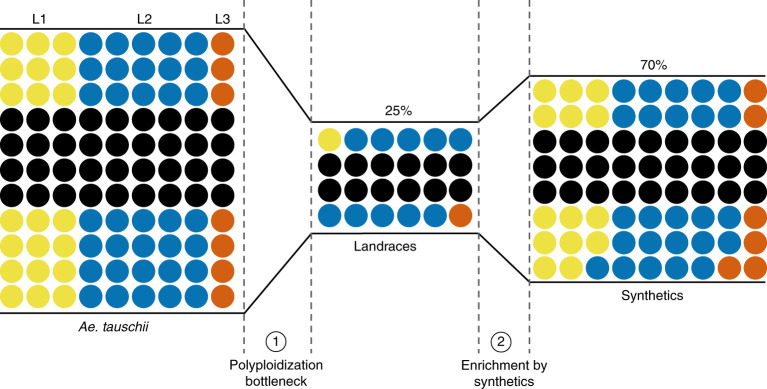
Restricted gene flow from *Ae. tauschii* to wheat and the capture of *Ae. tauschii* diversity in a panel of synthetic hexaploid wheats. Genetic diversity private to *Ae. tauschii* L1, L2 and L3 is color coded blue, red and orange, respectively, whereas black dots represent *k*-mer sequences (51-mers) common to more than one lineage. The number of dots is proportional to the number of *k*-mers. The polyploidization bottleneck (1) incorporated 25% of the variant *k*-mers found in *Ae. tauschii* into wheat landraces. The addition of 32 synthetic hexaploid wheats (2) restored this to 70%.

**Fig. 6:**
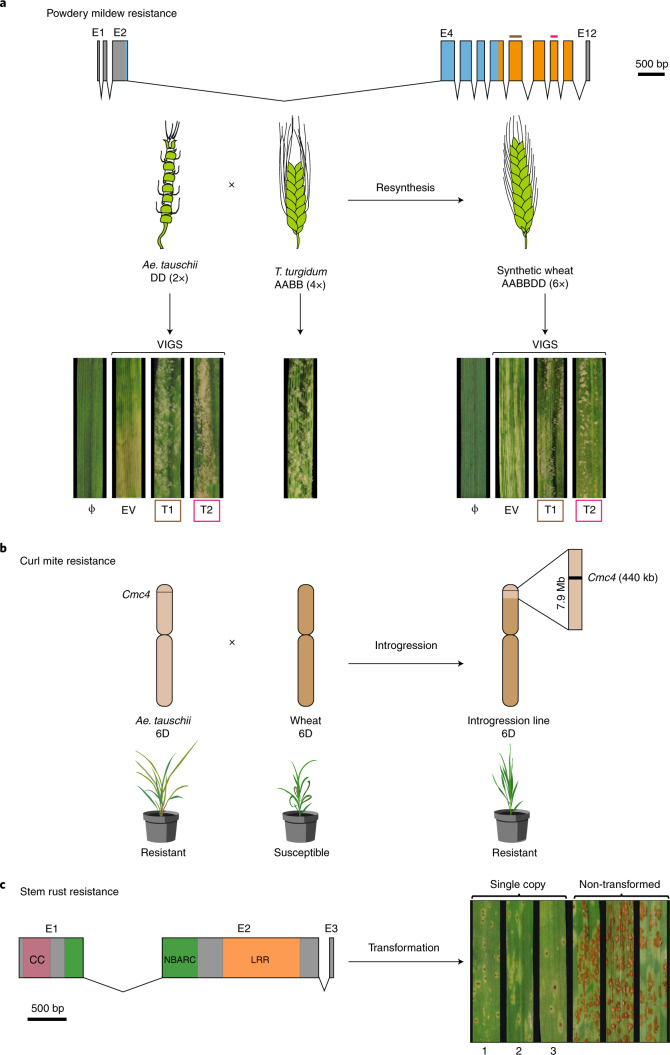
Functional transfer of disease and pest resistance from *Ae. tauschii* into wheat. **a**, *WTK4* gene structure represented by rectangles (exons E1 to E12) joined by lines (introns). Kinase domains are shown in blue and orange. Exons used for designing VIGS target 1 (T1) and target 2 (T2) are shown in brown and red, respectively. Below, schematic of the cross between *Ae. tauschii* accession Ent-079 (contains *WTK4*) and *T. turgidum* durum line Hoh-501 (lacks *WTK4*) that generated the synthetic hexaploid wheat line NIAB-144. Leaf segments from plants subjected to VIGS with empty vector (EV), T1, T2 or non-virus control (Φ) and super-infected with *B. graminis* f. sp. *tritici* isolate Bgt96224 avirulent to *WTK4*. **b**, Introgression of the *Cmc4* locus from *Ae. tauschii* accession TA1618 into wheat. The 440-kb *Cmc4* LD block (black) resides within a 7.9-Mb introgressed segment on chromosome 6D (light brown) in wheat cultivar TAM 115. Below, drawings of wheat curl mite-induced phenotypes. **c**, Structure of the *SrTA1662* candidate gene. The predicted 970-amino acid protein has domains with homology to coiled-coil (CC), nucleotide-binding (NB-ARC) and leucine-rich repeats (LRR). Right, transformation with an *SrTA1662* genomic construct into cv. Fielder and response to *P. graminis* f. sp. *tritici* isolate UK-01 (avirulent to *SrTA1662*) of single-copy hemizygous transformants (1, DPRM0059; 2, DPRM0051; 3, DPRM0071) and non-transgenic controls.

**Extended Data Fig. 9:**
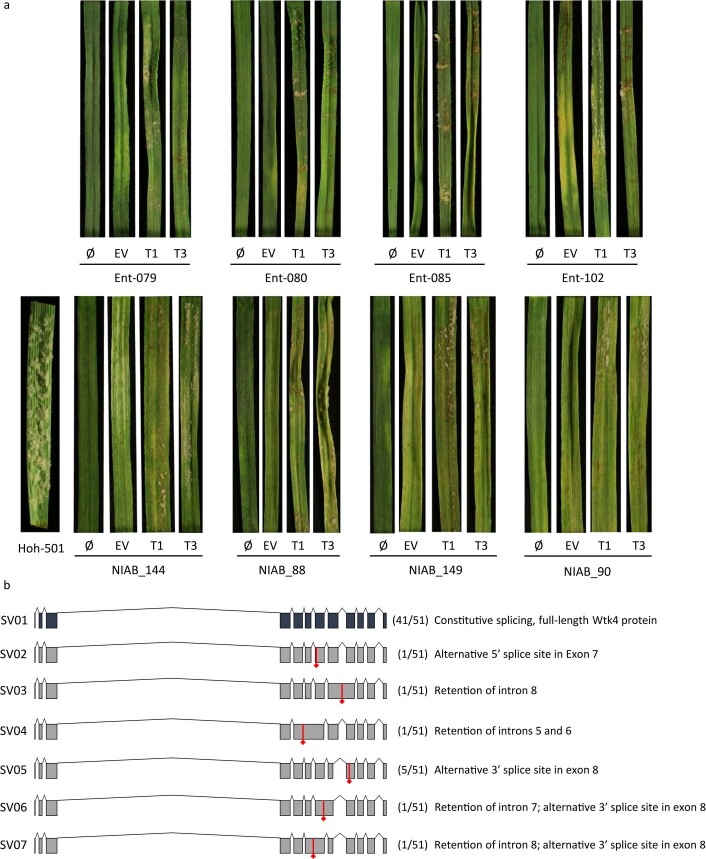
Analysis of powdery mildew resistance in *Aegilops tauschii* and durum donors and their derived synthetic hexaploid wheat lines. **a**, Top, disease reactions to *Blumeria graminis* f. sp. *tritici* Bgt96224 are displayed for the *Ae. tauchii* accessions Ent-079, Ent-080, Ent-085 and Ent-102. Bottom, disease reactions to Bgt96224 are displayed for the corresponding synthetic hexaploid lines (NIAB_144, derived from Ent-079; NIAB_088 derived from Ent-080; NIAB_149 derived from Ent-085; and NIAB_090 derived from Ent-102) using the tetraploid durum wheat donor line Hoh-501, which is highly susceptible to Bgt96224. Each *Ae. tauschii* and its corresponding synthetic hexaploid line was not inoculated with BSMV (Ø) or with a BSMV construct as empty vector (EV) or targeting for silencing the *WTK4* exon 8 (target 1, T1) or exon 10 (target 2, T2), respectively, and then super-infected with Bgt96224. **b**, Alternative splicing of *WTK4*. Alternative splicing variants (SV1-7) revealed by sequencing 51 *WTK4* cDNAs. At the top, in black, is shown the splicing variant SV01, which encodes the complete WTK4 protein. Below SV01, six aberrant alternative splicing variants (SV02 to SV07) are shown in in grey. The number of clones identified for each SV is identified in parenthesis. Diamond arrowed red lines point to the first stops codons at the protein level.

The ability to precisely identify *Ae. tauschii* haplotypes and candidate genes for target traits provides an opportunity for accelerating their introduction into cultivated wheat. We selected 32 non-redundant and genetically diverse *Ae. tauschii* accessions, which capture 70% of the genetic diversity across all lineages, and crossed them to tetraploid durum wheat (*T. turgidum* var. *durum*; AABB) to generate independent synthetic hexaploid wheat lines (Fig. 5, Supplementary Table 12 and Supplementary Note). From this ‘library’, we selected four synthetic lines with the powdery mildew *WTK* candidate resistance gene. These synthetics as well as their respective *Ae. tauschii* donors were resistant to powdery mildew, while the durum line was susceptible (Fig. 6a and Extended Data Fig. 9a). Annotation of *WTK* identified seven alternative transcripts, of which only one, accounting for ~80% of the transcripts, leads to a complete 2,160-base pair (bp) 12-exon open reading frame (Fig. 6a, Extended Data Fig. 9b, Supplementary Tables 13 and 14 and Supplementary Note). Next, we targeted two exons with very low homology to other genes for virus-induced gene silencing (VIGS; Supplementary Note). *WTK*-containing *Ae. tauschii* and synthetics inoculated with the *WTK*-VIGS constructs became susceptible to powdery mildew, whereas empty vector-inoculated plants remained resistant (Fig. 6a and Extended Data Fig. 9a). This supports the conclusion that *WTK*, hereafter designated *WTK4*, is required for powdery mildew resistance and remains effective in synthetic hexaploids. Thus, these synthetic lines can serve as prebreeding stocks for introduction of the trait into elite wheat.

Developing wheat cultivars improved with traits from *Ae. tauschii* can also be achieved by direct crossing between the diploid and hexaploid species^10^. The wheat curl mite resistance gene *Cmc4* was originally transferred by crossing of *Ae. tauschii* accession TA2397 (L1) into wheat^42,43^ and genetically localized to chromosome 6D in agreement with our association mapping^38–40^. Given the common resistant haplotype of *Cmc4* in L1 and L2 (Fig. 4), we hypothesized that *Cmc4* is the same as a gene originating from L2 accession TA1618, which was introgressed at the same locus into wheat cv. TAM 112 via a synthetic wheat^39,43^. Consistent with this hypothesis, we observed the same haplotype at the wheat curl mite resistance locus across all derived resistant hexaploid wheat lines and in the *Ae. tauschii* donors of *Cmc4* and *Cmc*_TAM112_ (Fig. 4c). We delimited the length of the introgressed *Ae. tauschii* wheat curl mite fragments by comparing SNP data for resistant wheat lines and the corresponding *Ae. tauschii* donors. The TA2397 (L1) introgression spanned 41.5 Mb, whereas the TA1618 (L2) introgression was reduced to 7.9 Mb in wheat cv. TAM 115 (Fig. 6b, Extended Data Fig. 7b,c and Supplementary Note).

**Extended Data Fig. 10:**
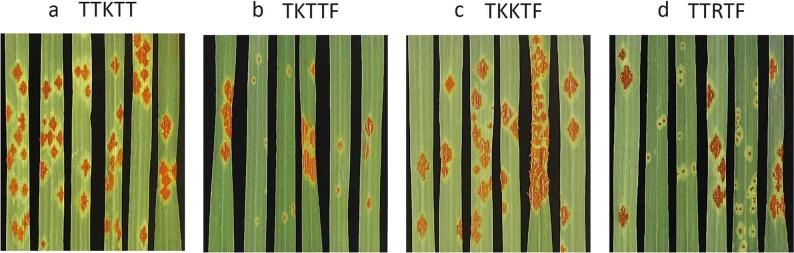
The *Aegilops tauschii* stem rust resistance gene *SrTA1662* maintains race specificity as a transgene in wheat. The *SrTA1662* gene was transformed into the stem rust susceptible wheat cultivar Fielder. Shown are T_2_ generation lines selected to be homozygous for the transgene or to be non-transgenic segregants. **a**, Inoculation with isolate IT200a/18 (race TKKTF). **b**, Inoculation with isolate IT16a/18 (race TTRTF). **c**, Inoculation with isolate ET11a/18 (TKTTF). **d**, Inoculation with isolate KE184a/18 (Kenya). Numbering refers to 1 = DPRM0050 (null of DPRM0051), 2 = DPRM0051, 3 = DPRM0059, 4 = DPRM0062 (null of DPRM0059), 5 = DPRM0071, 6 = DPRM0072 (= null of DPRM0071) (see Supplementary Table E).

As an alternative to conventional breeding, we targeted the *SrTA1662* candidate stem rust resistance gene (Fig. 2d) for introduction into wheat by direct transformation. We cloned a 10,541-bp genomic fragment encompassing the complete *SrTA1662* transcribed region as well as >3 kb of 3′- and 5′-untranslated region (UTR) putative regulatory sequences; this was sufficient to confer full race-specific stem rust resistance in transgenic wheat (Fig. 6c, Extended Data Fig. 10, Supplementary Table 15 and Supplementary Note).

## Discussion

The origin of hexaploid bread wheat has long been the subject of intense scrutiny. Archeological and genetic evidence suggests that diploid and tetraploid wheats were first cultivated 10,000 years ago in the Fertile Crescent (Fig. 1a)^5,6^. The expansion of tetraploid wheat cultivation northeast into Caspian Iran and towards the Caucasus region resulted in sympatry with *Ae. tauschii* and the emergence of hexaploid bread wheat^6^. *Ae tauschii* displays a high level of genetic differentiation among local populations, and genetic marker analysis suggests that the wheat D subgenome donor was recruited from an L2 population of *Ae. tauschii* in the southwestern coastal area of the Caspian Sea^8^. However, not all the diversity within the wheat D subgenome can be explained by a single hybridization event^6,44,45^. Our population genomic analysis revealed the existence of a third lineage of *Ae. tauschii*, L3, which also contributed to the extant wheat genome. For example, a glutenin allele required for superior dough quality was recently found to be of L3 origin^46^. L3 accessions are restricted to present-day Georgia and may represent a relict population from a glacial refugium as observed in *Arabidopsis*^47^. We observed genomic signatures specific to L2 and L3 in hexaploid wheat supporting the multiple hybridization hypothesis (Fig. 1g).

The creation of hexaploid bread wheat, while giving rise to a crop better adapted to a wider range of environments and end uses^1^, came at the cost of a pronounced genetic bottleneck^7^. Our analysis suggested that only 25% of the genetic diversity of *Ae. tauschii* contributed to the initial gene flow into hexaploid wheat (Fig. 5). To explore this diversity, we performed association mapping and discovered new gene candidates for disease and pest resistance and agromorphological traits underpinning abiotic stress tolerance and yield, exemplifying the potential of *Ae. tauschii* for wheat improvement (Fig. 6). We obtained discrete LD blocks of 50 to 520 kb, with the exception of flowering time, which resulted in a broad LD block of 5.5 Mb around the *FT1* locus (Figs. 2 and 3). The low degree of historical recombination around *FT1* is likely imposed by the reduced probability of intraspecies hybridization between populations carrying alleles promoting different flowering times. In contrast to the discrete mostly submegabase mapping intervals we obtained by association mapping with *k*-mer-based marker saturation, conventional biparental mapping studies on the D subgenome resulted in large intervals with a median of 10 Mb (Supplementary Table 16 and Supplementary Note).

In polyploid wheat, recessive variants are not readily observed; hence, genetics and genomics in wheat have mostly focused on rare dominant or semidominant variants^48^. Reflecting this, of 69 genes cloned in polyploid wheat by forward genetics, at least 62 have dominant or semidominant modes of action (Supplementary Table 17). This constraint is removed in *Ae. tauschii* by virtue of being diploid, which along with its rapid LD decay makes it an ideal platform for gene discovery by association mapping. Genes and allelic variants discovered in *Ae. tauschii* can subsequently be studied in wheat by generating transgenics or mutants or by using synthetic wheats. The first synthetic wheats were created in the middle of the last century by E. Sears and E. McFadden^49^, and since the late 1980s, synthetic wheats have been used extensively in breeding, for example, by the International Maize and Wheat Improvement Center (CIMMYT)^50^. However, without the use of high-resolution genomic information, the use of synthetic wheats was not precisely tracked. As illustrated here for wheat curl mite resistance, this led to the same gene being introgressed from two different *Ae. tauschii* lineages. Our study highlights how synthetic wheats can now be explored in a more directed manner. Our public library of synthetic wheats, which captures 70% of the diversity present across all three *Ae. tauschii* lineages, allows immediate trait assessment in a hexaploid background. The trait-associated haplotypes can be used to design molecular markers to precisely track the desired gene in a breeding program. In conclusion, our study provides an end-to-end pipeline for rapid and systematic exploration of the *Ae. tauschii* gene pool for improving modern bread wheat.

## Methods

### SNP calling relative to the AL8/78 reference genome

Following whole-genome shotgun sequencing, we called SNPs across the panel relative to the *Ae. tauschii* AL8/78 reference genome assembly. The 306 *Ae. tauschii* samples were aligned to the *Ae. tauschii* AL8/78 reference genome^14^ using HISAT2 default parameters^52^. All alignment BAM files were sorted and duplicates removed using SAMtools (v.1.9 ‘view’, ‘sort’ and ‘rmdup’ sub-commands). All BAM files were fed into the variant call pipeline using BCFtools (-q 20 -a DP,DV | call -mv -f GQ) with parallelization ‘-r $region’ of 4-Mb windows for a total of 1,010 intervals (regions). The raw variant files were filtered or recalled using a published AWK script based on DP/DV ratios (the ratio of non-reference read depth and total read depth) with default parameters (https://bitbucket.org/ipk_dg_public/vcf_filtering/src/master/) except minPresent parameter (we used minPresent = 0.8 and minPresent = 0.1). The minPresent=0.8 dataset was used for redundancy analysis. The minPresent = 0.1 and minPresent = 0.8 were both used for genome-wide association study (GWAS) analysis. The resulting matrix (104 million SNPs for minPresent = 0.1 concatenated using BCFtools v.1.11) were uploaded to Zenodo.

### Quality control for redundancy and residual heterogeneity

A total of 100,900 (100 every 4-Mb window) SNPs were randomly chosen to compute pairwise identity by state among all samples for a total of 46,665 comparisons using custom R and AWK scripts (https://github.com/wheatgenetics/owwc). For every sample pair, a percent identity greater than 99.5% was deemed redundant based on the histogram distribution of all identity by state values (Extended Data Fig. 1c). This analysis confirmed the results of the KASP analysis conducted on the L2 accessions (Extended Data Fig. 1b and Supplementary Note).

For each accession (except TOWWC0193, which is related to the reference genome AL8/78), the fraction of heterozygous SNPs in the total number of biallelic SNPs was computed. Based on the distribution of these values (Extended Data Fig. 1d and Supplementary Table 3), 0.1 was deemed to indicate a low degree of residual heterogeneity. BW_26042, with a value of 0.17, was found to be the only outlier exceeding this threshold.

Based on these quality control analyses, a non-redundant and genetically stable set of 242 accessions was retained for further analysis. The redundant pairs, along with the different similarity scores, are given in Supplementary Table 4, and the set of 242 non-redundant accessions is provided in Supplementary Table 5.

### De novo assembly from whole-genome shotgun short-read data

The primary sequence data of non-redundant accessions were trimmed using Trimmomatic v.0.238 and de novo assembled with the MEGAHIT v.1.1.3 assembler using default parameters^53^. The output of the assembler for each accession was a FASTA file containing all the contig sequences. The assemblies are available from Zenodo.

### Genome assembly of *Ae. tauschii* accession TOWWC0112

TOWWC0112 (line BW_01111) was assembled by combining paired-end and mate-pair sequencing reads using TRITEX^54^, an open-source computational workflow. A PCR-free 250-bp paired-end library with an insert size range of 400–500 bp was sequenced to a coverage of ~70. Mate-pair libraries MP3 and MP6, with insert size ranges of 2–4 kb and 5–7 kb, respectively, were sequenced to a coverage of ~20. The assembly generated had an N50 of 196 kb (Supplementary Table 7). The assembly is available from the electronic Data Archive Library (e!DAL).

### Genome assembly of *Ae. tauschii* accession TOWWC0106

Accession TOWWC0106 (line BW_01105) was sequenced on a PacBio Sequel II platform (Pacific Biosciences) with single-molecule, real-time chemistry and on the Illumina platform. For single-molecule, real-time library preparation, ~7 μg of high-quality genomic DNA was fragmented to a 20-kb target size and assessed on an Agilent 2100 Bioanalyzer^55^. The sheared DNA was end repaired, ligated to blunt-end adaptors and size selected. The libraries were sequenced by Berry Genomics. A standard Illumina protocol was followed to make libraries for PCR-free paired-end genome sequencing with ~1 μg of genomic DNA that was fragmented and size selected (350 bp) by agarose gel electrophoresis. The size-selected DNA fragments were end blunted, provided with an A-base overhang and then ligated to sequencing adapters. A total of 251.8 Gb of high-quality 150 paired-end PCR-free reads were generated and sequenced on the NovaSeq sequencing platform.

A set of 11.35 million PacBio long reads (289.6 Gb), representing a ~66-fold genome coverage, was assembled using the CANU pipeline with default parameters^56^. The assembled contigs were polished with 251.8 Gb of PCR-free reads using Pilon default parameters^57^. The resulting assembly had an N50 of 1.5 Mb (Supplementary Table 7). The assembly is available from e!DAL.

### Phenotyping the *Ae. tauschii* diversity panel and synthetic hexaploid wheat lines

#### Wheat stem rust

The wheat stem rust phenotypes with *P. graminis* f. sp. *tritici* isolate 04KEN156/04, race TTKSK, and isolate 75ND717C, race QTHJC, were obtained from Arora et al.^22^. As part of this study, we also phenotyped the same *Ae. tauschii* lines with isolate UK-01 (race TKTTF)^58^ (Supplementary Table 8) using the same procedures as described in ref. ^59^. UK-01 was obtained from Limagrain.

#### Trichomes

For counting trichomes and measuring flowering time in *Ae. tauschii*, 50 L1 accessions and 150 L2 accessions were pregerminated at ~4 °C in Petri dishes on wet filter paper for 2 d in the dark. They were transferred to room temperature (~20 °C) and daylight for 4 d. Three seedlings of each genotype were transplanted on 22 January 2019 into 96-cell trays filled with a mixture of peat and sand and then grown under natural vernalization in a glasshouse with no additional light source or heating at the John Innes Centre, Norwich, UK. Trichome phenotyping was conducted 1 month later. Close-up photographs of the second leaf from seedlings at the three-leaf stage were taken and visualized in ImageJ, and trichomes were counted along one side of a 20-mm leaf margin in the mid-leaf region. Measurements were taken from three biological replicates (Supplementary Table 8).

#### Flowering time, biological replicate 1

Three seedlings used for trichome phenotyping (see above) were transferred on 25 March into individual 2 l pots filled with cereal mix soil^60^. Flowering time was recorded when the first five spikes were three-fourths emerged from the flag leaf sheath, equivalent to a 55 on the Zadoks growth scale^61^ (Supplementary Table 8).

#### Flowering time, biological replicates 2 and 3

A total of 147 *Ae. tauschii* L2 accessions were grown in the winters of 2018/2019 and 2019/2020 in the greenhouse at the Department of Agrobiotechnology, University of Natural Resources and Life Sciences, Vienna, Austria. Seeds of each accession were sown in multitrays in a mixture of heat-sterilized compost and sand and stratified for 1 week before germination at 4 °C with a 12 h day/12 h night light regimen. Thereafter, the seeds were germinated at 22 °C and at the one-leaf stage vernalized for 11 weeks. Five seedlings per accession were transplanted to 4 l pots (18 cm in diameter, 21 cm in height) filled with a mixture of heat-sterilized compost, peat, sand and rock flour. In the winter of 2018/2019, one pot (= one replicate) per accession was planted, whereas in 2019/2020, two pots (= two replicates) were planted. The pots were randomly arranged in the greenhouse and maintained at a temperature of 14/10 °C day/night with a 12 h photoperiod for the first 40 d. At spike emergence, the temperature was increased to 22/18 °C day/night with a 16 h photoperiod at 15,000 lx. At least ten spikes per pot were evaluated for beginning of anthesis, taken as 60 on the Zadoks growth scale^61^, resulting in a minimum of 30 assessed spikes per accession. Flowering time was recorded every second day.

The flowering date was analyzed using a linear mixed model, which considered subsampling of individual spikes within each pot as follows:

$${{{\mathcal{Y}}}}_{ijkl} = \mu + g_i + e_j + ge_{ij} + r_{jk} + p_{ijk} + \varepsilon _{ijkl}$$

Here, ${{{\mathcal{Y}}}}_{ijkl}$ denotes the flowering date observation of the individual spikes, *μ* is the grand mean and *g*_*i*_ is the genetic effect of the *i*th accession. The environment effect, *e*_*j*_, is defined as the effect of the *j*th year, and the genotype-by-environment interaction is described by *ge*_*ij*_. *r*_*jk*_ is the effect of the *k*th replication within the *j*th year, *p*_*ijk*_ is the effect of the *i*th pot within the *k*th replication and *j*th year and *ε*_*ijkl*_ is the residual term. Analysis was performed with R v.3.5.1 (ref. ^62^) using the package sommer^63^ with all effects considered as random except *g*_*i*_, which was modeled as a fixed effect to obtain the best linear unbiased estimates (Supplementary Table 8).

#### Spikelets per spike

For *Ae. tauschii* spikelet phenotyping, 151 accessions from L2 were vernalized at a constant temperature of 4 °C for 8 weeks in a growth chamber (Conviron). After vernalization, the accessions were transplanted to 3.8 l pots in potting mix (peat moss and vermiculite) and placed in a temperature-controlled Conviron growth chamber with diurnal temperatures gradually changing from 12 °C at 02:00 to 17 °C at 14:00 with a 16 h photoperiod and 80% relative humidity. To represent biological replication, each accession was grown in two pots, and each pot contained two plants. At the transplanting stage, 10 g of a slow-release N-P-K fertilizer was added to each pot. At physiological maturity, 5–15 main stem/tiller spikes per replication (that is, per pot) were collected, and the number of immature as well as mature spikelets were counted. Any obvious weak heads from late-growing tillers were not included. Least square means for each replication were used for *k*-mer-based association genetic analysis (Supplementary Table 8).

#### Powdery mildew

Resistance to *B. graminis* f. sp. *tritici* was assessed with Bgt96224, a highly avirulent isolate from Switzerland^64^, using inoculation procedures previously described^65^. Disease levels were assessed 7–9 d after inoculation as one of five classes of host reactions: resistance (R; 0–10% of leaf area covered), intermediate resistance (IR; 10–25% of leaf area covered), intermediate (I; 25–50% of leaf area covered), intermediate susceptible (IS; 50–75% of leaf area covered) and susceptible (S; >75% of leaf area covered) (Supplementary Table 8).

#### Wheat curl mite

A total of 210 *Ae. tauschii* accessions, 102 from L1 and 108 from L2 (Supplementary Table 8), were screened for their response against wheat curl mite. *Aceria tosichella* (Keifer) biotype 1 colonies (courtesy of M. Smith, Department of Entomology, Kansas State University) were mass reared under controlled conditions at 24 °C in a 14 h light/10 h dark cycle using the susceptible wheat cv. Jagger. The biotype 1 colony was previously reported as avirulent toward all *Cmc* resistance genes^38,66–68^. A single colony consisted of an individual pot with ~50 seedlings, and 20 colonies were grown to have sufficient mite inoculum to conduct the phenotyping. Colonies were placed inside 45 cm × 45 cm × 75 cm mite-proof cages covered with a 36-µm mesh screen (ELKO Filtering Co.) to avoid contamination until being used to infest the *Ae. tauschii* accessions. Accessions from L1 and L2 were evaluated in independent experiments. Six plants per accession were individually grown in 5 cm × 5 cm × 5 cm pots under controlled conditions at 24 °C in a 14 h light/10 h dark cycle. Pots were arranged randomly in an incomplete block design where the block was the tray fitting 32 pots (8 rows and 4 columns). A single pot with the susceptible check cv. Jagger was included in each tray. Accessions were infested at the two-leaf stage, with mite colonies collected from infested pieces of leaves from the susceptible plants and spread as straw over the pots. Plants were evaluated individually 10–14 d after infestation. Wheat curl mite damage was assessed as curled or trapped leaves using a visual scale from 0 to 4, with 0 indicating no symptoms and 1 to 4 indicating increasing levels of curliness or trapped leaves (Extended Data Fig. 7a).

The adjusted mean or best linear unbiased estimator for each accession was calculated with the ‘lme4’ R package^69^ using the following linear regression model:

$$y_{ijkl} = \mu + G_i + T_j + R_{k(j)} + C_{l(j)} + e_{ijkl}$$

Here, *y*_*ijkl*_ is the phenotypic value, *µ* is the overall mean, *G*_*i*_ is the fixed effect of the *i*th﻿ accession (genotype), *T*_*j*_ is the random effect of the *j*th tray assumed as independent and identically distributed (iid) $T_j\approx N(0,\sigma _T^2)$, *R*_*k*(*j*)_ is the random effect of the *k*th row nested within the *j*th tray assumed distributed as iid $R_{k(j)}\approx N(0,\sigma _R^2)$, *C*_*l*(*j*)_ is the random effect of the *l*th column nested within the *j*th tray assumed distributed as iid $C_{l(j)}\approx N(0,\sigma _C^2)$ and *e*_*ijkl*_ is the residual error distributed as iid *e*_*ijkl*_ ≈ *N*(0, $\sigma _e^2$).

### *k*-mer presence/absence matrix

*k*-mers (*k* = 51) were counted in trimmed raw data per accession using Jellyfish^70^ (version 2.2.6 or above). *k*-mers with a count of less than two in an accession were discarded immediately. *k*-mer counts from all accessions were integrated to create a presence/absence matrix with one row per *k*-mer and one column per accession. The entries were reduced to 1 (presence) and 0 (absence). *k*-mers occurring in less than two accessions or in all but one accession were removed during the construction of the matrix. Programs to process the data were implemented in Python and are published at https://github.com/wheatgenetics/owwc. The *k*-mer matrix is available from e!DAL.

### Phylogenetic tree construction

A random set of 100,000 *k*-mers was extracted from the *k*-mer matrix to build an unweighted pair group method with arithmetic mean (UPGMA) tree with 100 bootstraps using the Bio.Phylo module from the Biopython v.1.77 (http://biopython.org) package. Further, a Python script was used to generate an iTOL-compatible (https://itol.embl.de/) tree for rendering and annotation. The Python script and the random set of 100,000 *k*-mers used for generating the tree are available at https://github.com/wheatgenetics/owwc.

### Bayesian cluster analysis using STRUCTURE

Bayesian clustering implemented in STRUCTURE^19^ version 2.3.4 was used to investigate the number of distinct lineages of *Ae. tauschii*. To control the bias due to the highly unbalanced proportion of the three groups^20^ in the non-redundant sequenced accessions (119 accessions of L2, 118 accessions of L1 and 5 accessions of putative L3), 10 accessions each of L1 and L2 were randomly selected for each STRUCTURE run along with the 5 accessions of the putative L3 and the control L1–L2 RIL. The random selection of 10 accessions each of L1 and L2 was performed 11 times without replacement, thus covering a total of 110 accessions each of L1 and L2 over 11 STRUCTURE runs (Supplementary Table 6). STRUCTURE simulations were run using a random set of 100,000 *k*-mers with a burn-in length of 100,000 iterations followed by 150,000 Markov chain Monte Carlo iterations for five replicates each of *K* ranging from 1 to 6. STRUCTURE output was uploaded to Structure Harvester (http://taylor0.biology.ucla.edu/structureHarvester; Web v.0.6.94 July 2014; Plot vA.1 November 2012; Core vA.2 July 2014)^71^ to generate a Δ*K* plot for each run. For each STRUCTURE run, a clear peak was observed at *K* = 3 in the Δ*K* plot, suggesting that there are three distinct lineages of *Ae. tauschii*^19,71^. STRUCTURE results were processed and plotted using CLUMPAK^72,73^ (http://clumpak.tau.ac.il/; beta version accessed on 11 May 2021) to maintain the label collinearity for multiple replicates of each *K*.

### Determination of genome-wide fixation index

Genome-wide pairwise fixation index (*F*_ST_) between the three *Ae. tauschii* lineages was computed using VCFtools^74^ v.0.1.15 with the parameters ‘–fst-window-size’ and ‘–fst-window-step’ set to 1,000,000 and 100,000, respectively.

### Admixture analysis of the wheat D subgenome

To assign segments of the wheat D subgenome to *Ae. tauschii* lineages for each of the 11 chromosome-scale wheat assemblies^21^, we considered only those *k*-mers as usable that were present at a single locus in the D subgenome. Furthermore, out of these *k*-mers, for nine modern cultivars, only those *k*-mers were considered usable that were also present in the short-read sequences from 28 hexaploid wheat landraces^17^. For the assembled wheat genomes, each chromosome of the D subgenome was divided into 100-kb non-overlapping segments. A 100-kb segment was assigned to *Ae. tauschii* if at least 20% of 100,000 *k*-mers within that segment were usable as well as present in at least one non-redundant *Ae. tauschii* accession. A segment assigned to *Ae. tauschii* was further assigned to one of the three lineages (L1, L2 and L3) if the count of usable *k*-mers specific to that lineage exceeded the count of those specific to the other lineages by at least 0.01% of 100,000 *k*-mers. Scripts to determine the counts of lineage-specific and total *Ae. tauschii k*-mers per 100-kb segment are published at https://github.com/wheatgenetics/owwc, and the output files obtained for 11 wheat assemblies were collated in an Excel file that is available from Zenodo.

### Anchoring of a de novo assembly to a reference genome

The contigs of a de novo assembly were ordered along a chromosome-level reference genome using minimap2 (ref. ^75^) (version 2.14 or above), and the genomic coordinates of their longest hits were assigned.

### Correlation prefiltering

For each of the assembly *k*-mers (including those present at multiple loci), if also present in the precalculated presence/absence matrix, Pearson’s correlation between the vector of that *k*-mer’s presence/absence and the vector of the phenotype scores was calculated. Only those *k*-mers for which the absolute value of correlation obtained was higher than a threshold (0.2 by default) were retained to reduce the computational burden of association mapping using linear regression.

### Linear regression model accounting for population structure

To each filtered *k*-mer from the previous step, a *P* value was assigned using linear regression with a number of leading PCA dimensions as covariates to control for the population structure. PCA was computed using the aforementioned set of 100,000 *k*-mers. The exact number of leading PCA dimensions was chosen heuristically. Too high a number might overcorrect for population structure, while too few might undercorrect. In the context of this study, three dimensions were found to represent a good trade-off.

### Approximate Bonferroni threshold computation

For each phenotype in this study, the total number of *k*-mers used in association mapping varied between 3,000,000,000 and 5,000,000,000. In general, if the *k*-mer size is 51, a SNP or any other structural variant would give rise to at least 51 *k*-mer variants. Therefore, the total number of tested *k*-mer variants should be divided by 51 to get the effective number of variants to adjust the *P* value threshold for multiple testing. Assuming a *P* value threshold of 0.05, a Bonferroni-adjusted –log *P* value threshold between 9.1 and 9.3 was obtained for each phenotype. The more stringent cutoff of 9.3 was chosen throughout this study.

### Generating association mapping plots

Association mapping plots were generated using Python. For a chromosome-level reference assembly, each integer on the *x* axis corresponds to a 10-kb genomic block starting from that position. For an anchored assembly, each integer on the *x* axis represents the scaffold that is anchored starting from that position. Dots on the plot represent the –log *P* values of the filtered *k*-mers within each block. Dot size is proportional to the number of *k*-mers with the specific –log *P* value. The plotting script is published at https://github.com/wheatgenetics/owwc.

### Optimization of *k*-mer GWAS in *Ae. tauschii*

We used previously generated stem rust phenotype data for *P. graminis* f. sp. *tritici* isolate 04KEN156/04, race TTKSK, on 142 *Ae. tauschii* L2 accessions^22^. Mapping *k*-mers with an association score of >6 to the *Ae. tauschii* reference genome AL8/78 gave rise to significant peaks for the positive controls *Sr45* and *Sr46* (Extended Data Fig. 4a). The peaks contain *k*-mers that are negatively correlated with resistance (shown as red dots) because the AL8/78 reference accession does not contain *Sr45* and *Sr46*. To identify the true *Sr45* and *Sr46* haplotypes, accession TOWWC0112 (which contains *Sr45* and *Sr46*)^22^ was assembled from tenfold whole-genome shotgun data using MEGAHIT (N50 = 1.1 kb) and used in association mapping. However, noise masked the positive signals from *Sr45* and *Sr46* when the short scaffolds were distributed randomly along the *x* axis (Extended Data Fig. 4b). Anchoring the scaffolds to the AL8/78 reference genome considerably improved the plot and produced positive signals for *Sr45* and *Sr46* (blue peaks; Extended Data Fig. 4c). An improved assembly (N50 = 196 kb), generated with mate-pair libraries and again anchored to AL8/78, further reduced the background noise (Extended Data Fig. 4d).

### Performing *k*-mer GWAS in *Ae. tauschii* with reduced coverage

The trimmed sequence data of each non-redundant accession was randomly subsampled to reduce the coverage to 7.5-fold, 5-fold, 3-fold and 1-fold. For each coverage point, the *k*-mer GWAS pipeline was applied, and *k*-mers with an association score of >6 were mapped to the *Ae. tauschii* reference genome AL8/78 (Extended Data Fig. 5).

### Computing genome-wide LD

The *Ae. tauschii* AL8/78 reference genome was partitioned into five segments (R1, R2a, C, R2b and R3; Extended Data Fig. 8) based on the distribution of the recombination rate, where the boundaries between these regions were imputed using the boundaries established for the Chinese Spring RefSeqv1.0 D subgenome^51^. PopLDdecay^76^ v.3.41 with the parameter ‘-MaxDist’ set to 5 Mb was used to determine the LD decay in these regions for both L1 and L2. For L2, the value of mean *r*^2^ in the telomeric regions R1 and R3 dropped below 0.1 at genomic distances of 291 kb and 476 kb, respectively, while for L1, the corresponding genomic distances were 661 kb and 561 kb, respectively.

### Reporting Summary

Further information on research design is available in the Nature Research Reporting Summary linked to this article

## Online content

Any methods, additional references, Nature Research reporting summaries, source data, extended data, supplementary information, acknowledgements, peer review information; details of author contributions and competing interests; and statements of data and code availability are available at 10.1038/s41587-021-01058-4.

## Supplementary information

Supplementary note

Reporting Summary

Supplementary Tables Supplementary Tables 1–17.

## Data Availability

The raw PacBio and Illumina sequences used for the assembly of *Ae. tauschii* accession TOWWC0106 have been submitted to the Genome Sequence Archive (GSA) of the National Genomics Data Center hosted by the Beijing Genomics Institute, Beijing, under the accession number CRA002681 and to NCBI under study number PRJNA730363. The genome assemblies and annotations of TOWWC0112 and TOWWC0106 are available from the Leibniz Institute of Plant Genetics and Crop Plant Research (IPK) at https://doi.ipk-gatersleben.de/DOI/4bb6f03f-3a15-429a-b542-9962cb676e63/953a2d8a-5ade-479a-9304-6fdd12da7ce4/2/1847940088. The 150-bp paired-end Illumina sequences for the 306 *Ae. tauschii* accessions, the 250-bp paired-end and mate-pair libraries for accession T0WW0112 and the RNA sequencing data for 8 *Ae. tauschii* accessions are available from NCBI, study number PRJNA685125. The 150-bp paired-end Illumina sequences for the hexaploid wheat accessions and the two additional *Ae. tauschii* accessions used in the *Cmc4* and *Cmc*_TAM112_ haplotype analysis (Fig. 4 and Extended Data Fig. 7b,c) are available from NCBI, study number PRJNA694980. The *k*-mer matrix for 305 *Ae. tauschii* accessions and the tetraploid donor *T. durum* Hoh-501 used to generate synthetic hexaploids can be obtained from https://doi.ipk-gatersleben.de/DOI/dfc2d351-b5fe-41e6-bd6c-efe96cfcc7aa/0cef0e89-acf2-451c-8efc-a71c0368fec4/2/1847940088. The variant call (SNP) file for 306 *Ae. tauschii* accessions based on the AL8/78 reference is available from Zenodo at 10.5281/zenodo.4317950. Counts of lineage-specific *k*-mers in wheat genome assemblies are available from Zenodo at 10.5281/zenodo.4474428. MEGAHIT assemblies for 303 *Ae. tauschii* accessions (including the 242 non-redundant accessions) are available from Zenodo at 10.5281/zenodo.4430803, 10.5281/zenodo.4430872 and 10.5281/zenodo.4430891. A 29,243-bp fragment extracted from contig 00015145 of the *Ae. tauschii* TOWWC0106 assembly was deposited in the NCBI GenBank along with the coordinates of the *WTK4* transcript SV01 under study number MW295405. The *SrTA1662* gene and transcript sequence have been deposited in NCBI Genbank under accession number MW526949. Figures that have associated raw data include Figs. 1–6 and Extended Data Figs. 1–9.

## Code availability

Scripts for SNP calling, *k*-mer matrix generation, redundancy analysis, determination of residual heterogeneity and phylogenetic tree construction, including iTOL.nwk files, admixture analysis, *k-*mer GWAS and SNP GWAS, can be found in the repository https://github.com/wheatgenetics/owwc.
