# Supplementary material for: Population genomic analysis of Aegilops tauschii identifies targets for bread wheat improvement
Source: Nat Biotechnol. 2021 Nov 1;40(3):422–31. doi: 10.1038/s41587-021-01058-4 (PMC8926922; doi:10.1038/s41587-021-01058-4)
Supplement: Supplementary file 1 — Supplementary note [file 41587_2021_1058_MOESM1_ESM.pdf]

nature portfolio

https://doi.org/10.1038/s41587-021-01058-4

Supplementary information

Population genomic analysis of *Aegilops tauschii* identifies targets for bread wheat improvement

In the format provided by the authors and unedited

Compilation of an inventory of *Ae. tauschii* accessions

We surveyed the literature, germplasm banks and unpublished collections to compile an inventory of 641 *Ae. tauschii* potentially unique accessions spanning its geographical range from western Turkey to eastern China. Accordingly, accessions of *Ae. tauschii* described by Singh *et al.* (2019) (549 accessions)[1], Arora *et al.*, (2019) (150 accessions)[2] and Abbasov *et al.* (2019) (36 accessions)[3] were cross-referenced to identify duplicates (accessions with the same name, or identical accessions with different names). Where available, missing collection site data from these lines was obtained from www.genesys-pgr.org. Within this set, genotyping-by-sequencing data[1] or records held at the Wheat Genetics Resource Centre, Kansas State University, USA, were used as further criteria to highlight genetically redundant lines. To this set, we added the geographic data from an unpublished collection of 81 accessions established by Ali Mehrabi at the Ilam University Gene Bank (IUGB). This collection includes 63 accessions collected across the moderate climatic ranges of the Zagros and Alborz mountains in northern and western Iran in 2006. Each sample was isolated based on a separation of at least 20 km or by geographical barriers. The spike samples from an area within a radius of 500 m were collected as one population (accession). Spikelet shape was used to identify subspecies, with intermediate forms considered as ssp. *tauschii*. Samples were bulked at Ilam University Research Farm, deposited to the Cereal Gene Bank at Ilam University, Īlām, Iran and assigned unique IUGB germplasm identification codes. An additional collection of 84 L1 accessions from Tajikistan, collected between 2005 and 2006 by Firuza Nasyrova, Institute of Botany, Plant Physiology and Genetics, Tajik National Academy of Sciences Tajikistan, was also included[4]. These accessions were assigned latitude and longitude coordinates based on the 13 geographical locations listed. In total, the inventory includes 641 accessions, of which passport data is available for 588 accessions (Supplementary Table 1; Extended Data Fig. 1a). Passport data for the sequenced D donors was also compiled as above, with further latitude and longitude data to that described in Arora *et al.* (2019) provided by Huw Jones, NIAB or obtained from www.genesys-pgr.org (Supplementary Table 12).

Selection of accessions for whole genome shotgun sequencing

Previously available KASP genotyping data from Arora *et al.* (2019)[2] was used to select a set of 24 genetically diverse *Ae. tauschii* accessions for short read Illumina sequencing with an average coverage of 30. The criteria used for selecting accessions included representation of different branches of a phylogenetic tree[2], country of origin, disease variation for six races of stem rust[5], and accessions carrying mapped and/or cloned resistance genes from *Ae. tauschii* (*Sr45*, *Sr33*, *Sr46*, *SrTA1662*, *Lr21* and *SrTA10171*). Of these 24 accessions, seven were from L1, sixteen from L2, and one from L3. Of the 171 accessions remaining in the diversity panel of the Arora *et al.* (2019) study[2], 130 L2 accessions were selected to maximize non-redundancy based on the histogram of similarity scores obtained from the KASP analysis (Extended Data Fig. 1b). A threshold of 97.5% separated the peak of high similarity values and was therefore taken to be the redundancy cut-off. Notwithstanding this cut-off, among the 130 selected accessions, 28

accessions were above the redundancy cut-off but were included due to secondary considerations including disease variation for stem rust races and seed availability. These 130 accessions were targeted for whole genome shotgun (WGS) sequencing with an average coverage of 10.

To cover the genetic diversity of L1, we selected 110 putative L1 accessions based on the analysis performed by Singh *et al.* (2019)[1]. These accessions were also targeted for WGS sequencing at 10-fold genome coverage. Two of these accessions (TOWWC0253, line BW_23897 and TOWWC0304, line BW_23925) were subsequently found to belong to L2 after analysis of the sequencing data.

After discovering the distinct signature of L3 in wheat, 10 putative inter-lineage hybrids from Singh *et al*. (2019)[1] were sequenced with an average coverage of 7.5-fold genome coverage. Seven of these putative hybrids were found to belong to L3, one was found to belong to L1, and, as previously determined by Singh et al. (2019)[1], two were recent (possibly, post collection) recombinant inbred lines of L1 and L2.

We also sequenced 40 *Ae. tauschii* donors used to create synthetic hexaploid wheats with the durum wheat cultivar Hoh-501 (see below). Eleven of these donors were included in the above selections. The remaining 29 were sequenced with an average coverage of 7.5-fold.

In addition, we sequenced accessions TA1618 and TA2394, which were used for introgression of *Cmc4* into wheat, giving a tally of 306 sequenced accessions which were used for subsequent analyses. Separately, we included two more L2 accessions TA2450 and Clae 23, the former because of the strategic importance as the parent of a TILLING population[6]. Based on the GBS and KASP analyses, respectively, referred to above, these accessions are redundant in the panel (Supplementary Table 1).

DNA extraction and short-read WGS-sequencing

DNA from accessions TOWWC0001 to TOWWC0195 was made available from Arora *et al.* (2019)[2]. For accessions TOWWC0196 to TOWW0304, the SHW lines and their diploid and tetraploid donors, high molecular weight DNA was extracted following a modified CTAB protocol[7]. For accessions TOWW0196 to TOWW0304 the same plant was used for DNA and single seed descent. Accessions TOWWC0001 to TOWW0304, and the SHW diploid and tetraploid donors, were sequenced on an Illumina platform with 150 bp PE chemistry at Novogene (China). The targeted sequencing coverage ranged from 7-fold (39 accessions), to 10-fold (234 accessions) to 30-fold (24 accessions) (Supplementary Table 2).

RNA extraction and RNAseq

Total RNA was obtained from accessions TOWWC0020 (line BW_20689), TOWWC0104 (line BW_21074), TOWWC0106 (line BW_21086), TOWWC0107 (line BW_21090), TOWWC0108 (line BW_21096), TOWWC0112 (line BW_21114), TOWWC0134 (line BW_21215) and TA2450. Seeds were germinated in a Petri dish at 22 °C in the dark in the presence of 0.5 ppm gibberellic acid and then transferred to a cereal soil mix[8] and placed in a controlled environment room at 18 °C with a 16 hour light/ 8 hour dark cycle. Leaf tissue from two-week old seedlings was harvested in the afternoon and immediately used for RNA extraction using a Qiagen RNA extraction kit followed by bead size selection. The RNA was used to generate Illumina TruSeq libraries which were sequenced on an Illumina platform with 150 bp paired end reads to generate the following amounts of data per accession: TOWWC0020, 34.98 Gb; TOWWC0104, 12.58 Gb; TOWWC0106, 34.47 Gb; TOWWC0107, 12.46 Gb; TOWWC0108, 35.36 Gb; TOWWC0112, 38.28 Gb; TOWWC0134, 33 Gb; TA2450, 35 Gb.

Annotation of the TOWWC0112 and TOWWC0106 genome assemblies

Structural gene annotations for TOWWC0112 (line BW_01111) and TOWWC0106 (line BW_01105) were done combining two annotation strategies: prediction based on comparative *ab initio* gene finding and a lift-over approach. The comparative *ab initio* approach warrants identification of gene model variations, while ensuring uniformity between the annotated genomes. To counteract any further intrinsic error during the *ab initio* gene calling, a consolidation step by projecting the existing *Ae. tauschii* gene models onto the BW_01111 and BW_01105 scaffolds was applied.

Comparative *ab initio* gene prediction resorts to utilising whole-genome sequence alignments (WGA). So, a WGA between TOWWC0112, TOWWC0106 and *Ae. tauschii*[9] using the cactus pipeline (Version 1.0)[10] was constructed. Prior to the alignment step, all nucleotide sequences were 20-kmer-softmasked to reduce complexity and facilitate construction of the WGA using the tallymer subtools from the genome tools package (Version 1.6.1)[11]. The resulting WGA was used as an input for the Augustus comparative annotation pipeline (Version 3.3.3)[12]. Extrinsic hints for intron and CDS regions were generated from the *Ae. tauschii* annotation in order to guide gene calling and reduce false positive predictions. Furthermore, a previously trained specific Aegilops model was used.

Predicted gene models were subsequently classified into high- or low-confidence. Non-redundant candidate protein sequences were compared against the following three manually curated databases using BLASTp (Version 2.3.0+): first, PTREP (http://botserv2.uzh.ch/kelldata/trep-db/index.html (Release 19)), a database of hypothetical proteins that contains deduced amino acid sequences in which, in many cases, frameshifts have been removed, which is useful for the identification of divergent TEs having no significant similarity at the DNA level; second, UniPoa, a database comprised of annotated Poaceae proteins; third, UniMag, a database of validated magnoliophyta proteins. UniPoa and UniMag protein sequences were downloaded from Uniprot

and further filtered for complete sequences with start and stop codons. Best hits were selected for each predicted protein to each of the three databases. Only hits with an E-value below 10e-10 were considered. A high confidence protein sequence is complete and has a subject and query coverage above the set threshold of 66% in the UniMag database, or no blast hit in UniMag but in UniPoa and not TREP. A low confidence protein sequence is not complete and has a hit in the UniMag or UniPoa database but not in TREP, or no hit in UniMag and UniPoa and TREP but the protein sequence is complete. The UniPoa, UniMag and UniProt databases were accessed from https://www.uniprot.org (Release 2016_07, downloaded: 3 Aug 2016).

For a consistent annotation between BW_01111, BW_01105 and *Ae. tauschii* AL8/78, gene models from *Ae. tauschii* AL8/78 were additionally projected onto the BW_01111 and BW_01105 sequences using a lift-over approach as previously described in[13]. Afterwards, high-confidence *ab initio* and projected gene models were combined. In case of overlap, the *ab initio* gene model was retained. The resulting merger was again confidence-classified using the methods described above.

On top of this homology-based classification, further tweaking was done relying on functional assignment. First, functional annotations of predicted protein sequences were generated using the AHRD pipeline (https://github.com/groupschoof/AHRD). Second, human-readable description lines were scanned for TE, plastid and non-protein coding keywords and gene models were tagged accordingly. Any non-tagged low-confidence proteins with an AHRD 3*-rating were promoted to high-confidence. Contrarily, high-confidence proteins were demoted if their AHRD rating was only a one star. An overview of the number of high confidence and low confidence annotated genes can be found in Supplementary Table 9.

Completeness of the predicted gene space was measured with BUSCO (version 4.06, viridiplantae orthodb10)[14] (Supplementary Table 9). The annotations are available from e!DAL.

Delineation of the *Cmc4* region

We performed alignment (bowtie2 v2.2.9 –end-to-end –very-fast) of WGS data to reference AL8/78 and performed variant calling using BCFtools (v1.9) as described above in the genetic redundancy section. We then conducted SNP-based association mapping with Q+K model using GAPIT (10.1093/bioinformatics/bts444) for the wheat curl mite trait. The region was preliminarily determined to be 2.1-2.6 Mb (chr6D) based on a p-value cut-off of 3.7e-7 (FDR = 0.05). We then constructed a more diverse panel including hexaploid wheat (with known wheat curl mite response) to further refine the region and infer allele introgressions. WGS sequence data for 121 entries, 36 wheat lines and a subset of 85 *Ae. tauschii* accessions, 25 from L1 (14 resistant and 11 susceptible) and 60 from L2 (six resistant and 54 susceptible), were aligned to an *in silico* synthetic reference genome created by combining the A and B genomes from the hexaploid wheat cv.

Jagger[15] and the *Ae. tauschii* genome assembly (Aet v4.0; NCBI BioProject PRJNA341983) as the D genome. The *Ae. tauschii* accessions were selected to include all the accessions with conclusive resistant (WCM infestation < 0.07) and susceptible phenotypes (WCM infestation > 3) (Supplementary Table 11). The alignment steps (HISAT2, v2.1.0) and variant calls are the same as described for the genetic redundancy section. To obtain high-quality SNP markers, VCF files were filtered by read depth (DP>=4) and quality (QUAL >30) using BCFtools v1.9[16], and for minor allele frequency (MAF>=0.1), missing data (minPresent >15%), and heterozygosity (het percent < 5%) using a customized pipeline. Retained SNP markers were 947,937 for chromosome 6DS (0 – 230 Mb) and 3,122 for the WCM resistance interval (1.9 – 2.7 Mb, with an extra 0.1 – to 1.2 Mb flanking based on the region identified above).

Delimiting introgressions with curl mite resistance in wheat

To delimit the extent of the *Ae. tauschii* introgressions conferring resistance to wheat curl mite into hexaploid wheat we manually explored SNP polymorphisms using whole genome shotgun data for four wheat curl mite resistant wheat lines, 'KS96WGRC40', 'TAM 112', 'TAM 115', and 'TAM 204', and two *Ae. tauschii* resistant donor accessions, TA2397 and TA1618 (Extended Data Fig. 7b-c). The variant calling (BCFtools) and filtering steps are the same as described above. The 'KS96WGRC40' resistance donor is the L1 accession TA2397 and the original line where *Cmc4* was mapped[17,18]. The 'TAM 112' resistance donor is the L2 accession TA1618 (PI-268210) through the cultivar 'Largo', and the line where Cmc_{TAM112} was mapped[19,20]. 'TAM 115' and 'TAM 204'[21] are both resistant through 'TAM 112'. Of particular note is that both wheat lines 'KS96WGRC40' and 'TAM 112' also carry the *Ae. tauschii* L2 accession TA2460 (BW_01148), susceptible to WCM.

Gene structure and alternative splicing of *WTK4*

A first *in silico* annotation of the *WTK4* gene was done based on RNAseq data from the *Ae. tauschii* accession TOWWC0107 (line BW_01106). We used the SMARTer™ RACE cDNA Amplification Kit (634923, Clontech) to determine the 5'- and 3'–ends using *WTK4*-specific primers (Supplementary Table 13) on cDNA generated from TOWWC0112 mRNA, which was extracted with the Dynabeads™ mRNA DIRECT™ Purification Kit (61012, Invitrogen). For reverse transcription of cDNA, the 3' SMART CDS Primer II A was replaced by primer GH438 in the 5' RT reaction. Subsequently, the same reaction containing the tailed first strand cDNA could be used for both 3' and 5' race PCR. 5' RACE PCR was performed with 2 µl of 1:5 diluted cDNA in a 20 µl reaction with the KAPA2G Robust PCR Kit (KK5501, Sigma-Aldrich, St. Louis, Missouri, USA) buffer B, gene specific reverse primer JS633 and the UPM primer provided with the Kit. 30 cycles were run according to the touchdown PCR program 1 described in the SMARTer™ RACE Kit manual. The 3' race PCR was made with 4 µl of 1:5 diluted cDNA in a 20 µl reaction with the KAPA2G Robust PCR Kit and buffer B, gene specific forward primer JS655 and the universal reverse primer GH439. After initial denaturation at 95°C for 3 min, a

touchdown PCR protocol with 10 cycles of 95°C for 15 s, 68°C (-0.8°C/cycle) for 30 s, 72°C for 30 s, then 25 cycles at 95°C for 15 s, 61°C for 15 s, 72° for 30 s was performed with a final extension at 72° C for 5 min. 3' and 5' race PCR fragments were subjected to agarose gel electrophoresis, excised, cloned and then sequenced by Sanger sequencing to determine the UTRs. Based on 5' RACE reactions, we could confirm the presence of at least 82 bp of 5' UTR without alternative start codons and a 3' UTR of at least 155 bp.

Guided by the 5' and 3' UTRs, we designed primers located on both UTRs to study gene structure and splicing. *WTK4* transcript accumulation was confirmed by PCR amplification using the primers JS693xJS671 followed by a semi-nested reaction with primers JS696xJS671 using KAPA Hifi HotStart Polymerase (KK2502, Kapa Biosystems, Hoffmann-La Roche) with an annealing temperature of 60°C and extension time of 3 min. PCR products were subcloned using CloneJET PCR Cloning Kit (K1232, Thermo Fischer Scientific, Waltham, Massachusetts, USA), according to the manufacturer's recommendations, and transformed into *E. coli*. Fifty-one single-colony derived plasmid clones were sequenced with the internal primers JS655, JS656, JS661, JS678, JS682, JS689, JS833 and JS835 (Supplementary Table 13). Transcript 1, which encodes the complete WTK4 protein, was found to be the most common transcript (80%), while the other six isoforms are less abundant (between 2% and 10%) and encode truncated proteins (Extended Data Fig. 15; Supplementary Table 14). Core kinase domains were predicted based on the Conserved Domain Database (CDD) from NCBI[22].

Virus-induced gene silencing of *WTK4*

To minimize the possibility of off-target silencing, we blasted the *WTK4* coding sequence against the reference genome assemblies of wheat cv. Chinese Spring[23] and *Ae. tauschii*[9] for the selection of *WTK4* gene fragments of 150-250 bp with no homology to other genes. Primers JS657xJS658 and JS662x663, with *Not*I and *Pac*I restriction sites in antisense direction to enable antisense insertion in the pBS-BSMV-γ vector, were used to amplify WTK4_target_1 (Exon 10) and WTK4_target_2 (Exon 8), respectively. Seeds from selected *Ae. tauschii* and synthetic hexaploid wheat accessions were stratified at 4°C for five days and then placed in a growth chamber (Conviron, Winnipeg, Canada) cycled at 23°C/16°C, 16/8 hour photoperiod with 60% humidity and a light intensity regime of 350 μmol/s·m^2. An equimolar amount of pBS-BSMV-α, pBS-BSMV-β and pBS-BSMV-γ transcripts carrying WTK4_target_1 or WTK4_target_2, was inoculated into fully-expanded first leaves of selected *Ae. tauschii* and synthetic hexaploid wheat accessions, and the durum donor Hoh-501, as previously described[24,25,26]. For *in vitro* synthesis of viral RNA, the Invitrogen™ mMESSAGE mMACHINE™ T7 Transcription Kit (Thermo Fischer Scientific, Waltham, Massachusetts, USA) was used according to the manufacturer's recommendations. 14 days after virus infection, 3rd and 4th leaves were detached, placed on 0.5% agar plates complemented with 10 g/L Benzylaminopurine and infected with *Bgt96224*. Powdery mildew phenotypes were visually assessed 7-10 days after infection (Extended Data Fig. 9a).

Gene structure of *SrTA1662* and protein domain annotation

The *SrTA1662* gene was assembled using overlapping contigs from assemblies of *Ae. tauschii* accessions TOWWC0107 (line BW_01106) and TOWWC0050 (line BW_01049) to 10,445 bp. This sequence was confirmed by Sanger-sequencing of overlapping PCR fragments generated from genomic DNA. The intron-exon structure of the gene was predicted by mapping the RNAseq data from TOWWC0107 (line BW_01106). The gene was annotated to have three exons that encode a 970 amino acid protein with a CC and NB-ARC domain and 14 LRRs predicted by NCBI and Pfam databases and LRRpredictor[27] (Fig. 6c).

Engineering of *SrTA1662* binary construct for transformation

A 10,455 bp fragment encompassing 3,395 bp of putative 5' regulatory sequence, 4,026 bp from the ATG to the STOP, and 3,0724 bp of putative 3' regulatory sequence, and flanked by synthetic 5' *Not*I and 3' *Pme*I sites, respectively, was synthesized by Thermofisher Scientific. The DNA fragment was cloned into pMA to yield the toolkit vector 18ACBRCC_SRTA1662_pMA, available from Addgene (#154372). The GoldenGate (MoClo) compatible level 2 vector pGoldenGreenGate-M (pGGG-M)[28] was modified to receive this fragment. Initially, a vector was assembled with the hygromycin phosphotransferase (*hpt*) gene with the castor bean catalase (*CAT-1*) intron driven by the rice *Actin1* promoter and an adaptor at MoClo position 2 which contained the unique restriction enzyme sites *Not*I and *Pme*I. In brief, the Level 1 construct pICH47802-RActpro::HptInt::NosT (selectable maker) and the Postion 2 *Not*I/*Pme*I adaptor were cloned into the binary level 2 vector pGGG-M using standard Golden Gate MoClo assembly[29]. The resulting vector was deemed pGGG-AH-*Not*I/*Pme*I. The native promoter, coding region and 3' UTR of *SrTA1662* was cloned as one 10,461 bp fragment into the *Not*I and *Pme*I sites of pGGG-AH-*Not*I/*Pme*I using standard cloning procedures, resulting in the final wheat transformation vector pGGG-AH-SrTA1662. The construct was electroporated into the hypervirulent *Agrobacterium tumefaciens* (strain AGL1)[30] in addition to the helper plasmid pAL155 which contains an additional *Vir*G gene. *Agrobacterium*-standard inoculums[31] were prepared as previously described[28].

Wheat transformation

The hexaploid wheat cv. Fielder, which is susceptible to *P. graminis* f. sp. tritici isolate UK-01[32], was transformed as previously described[28]. Under aseptic conditions wheat immature embryos were isolated, pre-treated by centrifugation, inoculated with *Agrobacterium* AGL1 containing pGGG-AH-SrTA1662 and co-cultivated for 3 days. Wheat callus induction and proliferation, shoot regeneration and rooting were carried out under a stringent hygromycin selection regime before the regenerated plantlets were transferred from *in vitro* to soil and acclimatized to ambient conditions. Transgenesis and transgene copy number analysis was performed by iDNA Genetics, Norwich, UK using Taqman qPCR and the *hpt* probebar[28,33]. Homozygous single-copy T_2 lines

and their respective nulls are available from the Germplasm Resources Unit www.SeedStor.ac.uk under entry numbers DPRM0050 to DPRM0074 (Supplementary Table 15).

Phenotyping *SrTA1662* transgenics for stem rust resistance

Primary (T_0) hemizygous transgenic wheat plants and non-transgenic controls, which had also undergone tissue culture, were phenotyped with *Puccinia graminis* f. sp. tritici isolate UK-01 as previously described[34], and the infection phenotypes were correlated with the presence of the *hpt* transgene (Fig. 6c; Supplementary Table 15).

To test for race-specificity, three independent T_2 homozygous lines and their respective non-transgenic segregants (nulls) were phenotyped with four phylogenetically distinct isolates of stem rust from Clade I (isolate KE184a/18, also known as Ug99, race TTKTT), Clade IV-B (isolate ET11a/18, race TKTTF), Clade IV-F (isolate, IT200a/18, race TKKTF), and Clade III-B (isolate IT16a/18, race TTRTF) according to nomenclature implemented by the Global Rust Reference Center (GRRC), Denmark (Extended Data Fig. 10; Supplementary Table 15). The macroscopic phenotype of the lines was investigated on the 1st and 2nd leaf at the seedling stage in a quarantine greenhouse at the GRRC. Four seedlings of each line were grown in peat moss at 20°C for 11 days in two replicates. Urediniospores of the isolates were retrieved from -80°C storage, heat-shocked at 43°C for 5 minutes, and then suspended in light industrial mineral oil (Novec 7200) prior to spray inoculation of plants on day 11. Inoculated plants were incubated in a humid chamber at 18±2°C in darkness overnight and then maintained in a glasshouse at 20±2°C with 16 hour light/ 8 hour darkness. Qualitative infection types (IT) for individual lines and isolates were assessed 15 days post inoculation using a standard 0-4 scale[35,36]. Infection types up to 3- were considered incompatible ('resistant' host), while 3+ and 4 represented a compatible interaction ('susceptible' host).

Creation of synthetic hexaploid wheat lines

A collection of 429 *Ae. tauschii* accessions representing 14 geographic regions was sourced from seven international germplasm collections. Each accession was genotyped using 15 D genome specific microsatellite simple sequence repeat (SSR) markers covering the entire D genome except the 7D chromosome[37]. A subset of 232 accessions were further genotyped at 62 D genome specific SNP loci identified by the UK wheat SNP consortium[38,39]. A targeted set of 100 individuals were selected which captured both the geographic distribution and genetic diversity of the wider collection to use as D genome donors in a synthetic resynthesis programme. Hybridization compatibility with the tetraploid donors meant we were able to produce fertile hexaploid synthetic lines from 52 of the selected *Ae. tauschii* accessions. Forty-three of these lines were made with the *T. turgidum* var. durum line Hoh-501 (a winter durum line obtained from Friedrich Longin, University of Hohenheim, Germany) and have been described as part of this study.

Seeds of the selected *Ae. tauschii* accessions and the durum line Hoh-501 were sown directly into Levington's M2 compost and germinated in a controlled glasshouse with a 16 hour photoperiod at 20°C and an 8 hour dark period at 15°C. Once germinated, all seedlings were moved to a vernalization chamber for 8 weeks with a 10 hour photoperiod and 14 hour dark period at 4°C. Following vernalization, all seedlings were replanted into 1 litre pots in course nutrient-rich compost, transferred to the glasshouse and grown under the same conditions as previously.

Durum Hoh-501 spikes were emasculated between growth stages 55 and 59 on the Zadoks scale[40] when the spike was between 50% and full emergence from the flag leaf. Less mature florets were removed so that all remaining florets were at a similar developmental stage. Florets were cut to around half their size to allow removal of all immature green anthers. Once anthers had been removed, the emasculated ear was covered with a clear glassine bag to avoid desiccation and cross-pollination. Two or three days post emasculation, *Ae. tauschii* plants with extruding pollen were selected as pollen donors for artificial pollination of receptive Hoh-501 stigmas.

At 3 weeks post pollination, all set seed were removed from the recipient female ear and cleaned in 20% bleach solution for 15 minutes. The embryo of each seed was excised under aseptic conditions and placed on Murashige and Skoog media with 20 g/l sucrose, 2 mg/l zeatin, 2.5 mg/l CuSO4, 6 g/l Sigma type 1 agarose, at pH 5.8. The embryo was placed with the scutellum in contact with the media and the Petri dish sealed with parafilm. Excised embryos were placed on covered trays in a growth room held at 25°C for 1-2 weeks until germination. Embryos that failed to germinate within two weeks were given a cold treatment of 4°C for seven days to encourage germination. Any embryos that failed to germinate after cold treatment were discarded. Germinated plantlets were then vernalized for 8 weeks at 4°C with a 10 hour photoperiod. After vernalization, plantlets were transferred to 1 litre pots containing Levingtons M2 compost and placed in a growth chamber at 20°C with a 16 hour photoperiod and 8 hour dark period at 15°C until they reached growth stage 23 on the Zadoks scale[40].

At this stage after transplantation the plants were treated with colchicine to double their chromosome complement. A solution of 0.05% colchicine was prepared using 12 ml distilled water, 6 mg colchicine, 0.18 ml dimethyl sulfoxide and a single drop of Tween-20 per plant. Strong tillering (4-6 leaves) haploid seedlings were removed from their pots and excess soil washed from the roots under a running tap. The roots were trimmed to around 2-3 cm and the leaves trimmed to a height of 10-15 cm. The roots of the seedlings were immersed in a beaker containing 13 ml of the pre-prepared 0.05% colchicine solution. A clear plastic bag was placed over the seedlings and two angle-poise lamps were placed either side of the transparent bag to encourage transpiration and therefore the uptake of colchicine through the seedlings. After 5.5 hours of exposure, the seedlings were removed from the colchicine solution and the roots were washed thoroughly under a running tap. The seedlings were potted into moist Levingtons M2 compost and transferred to a growth chamber held at 20°C and a 16 hour photoperiod. The treated seedling will often die back

within 1–2 weeks of colchicine treatment and new shoots will emerge shortly afterwards that can be grown to maturity. All emerging ears were bagged and seed of the primary synthetic wheat collected at maturity. The lines are available from the Germplasm Resources Unit www.SeedStor.ac.uk under entry numbers WS0461 to WS0501 (*Ae. tauschii* donor lines), WS0001 to WS0043 (synthetic hexaploid wheat lines) and WS0502 (durum line Hoh-501) (Supplementary Table 12).

Analysis of genetic redundancy based on whole genome shotgun sequencing (see above) subsequently revealed that some of the *Ae. tauschii* donor accessions were genetically redundant with each other. Moreover, we were unable to germinate the seed for harvesting tissue for DNA preps and sequencing for four *Ae. tauschii* donors. Our final set therefore included 32 sequenced and unique non-redundant *Ae. tauschii* donor accessions (Supplementary Table 12).

Determining gene-mapping interval sizes in the D genome

The 2017 Komugi wheat gene index (https://shigen.nig.ac.jp/wheat/komugi/genes/symbolClassList.jsp) was consulted to catalogue designated genes present within the genome of *Ae. tauschii* or the D-subgenome of *T. aestivum* and which had been mapped using bi-parental genetics. Candidate genes were filtered based on the existence of the following information within references listed in the Komugi index; (i) the presence of a marker either side of each gene, (ii) the size of the bi-parental population, and (iii) the marker number. The forward and reverse primer sequences for each marker were obtained from GrainGenes (https://wheat.pw.usda.gov/GG3/) or from published literature and BLASTed against the wheat cv. Chinese Spring assembly (IWGSC, INSDC GCA 900519105.1), available at EnsemblPlants. The best candidate location for each marker was used to calculate the mapping interval for each gene (Supplementary Table 16).

References

1 Singh, N. *et al.* Efficient curation of genebanks using next generation sequencing reveals substantial duplication of germplasm accessions. *Sci. Rep.* **9**, (2019).

2 Arora, S. *et al.* Resistance gene cloning from a wild crop relative by sequence capture and association genetics. *Nat. Biotechnol.* **37**, 139–143 (2019).

3 Abbasov, M. *et al.* Genetic diversity of *Aegilops* L. species from Azerbaijan and Georgia using SSR markers. *Genet. Resour. Crop Evol.* **66**, 453–463 (2019).

4 Nasyrova, F. Y. *Polymorphism and the genetic diversity of wheat and their relatives in Tajikistan*. (Publishing House Irfon, 2020).

5 Rouse, M. N., Olson, E. L., Gill, B. S., Pumphrey, M. O. & Jin, Y. Stem rust resistance in

Aegilops tauschii germplasm. *Crop Sci.* **51**, 2074–2078 (2011).

6 Rawat, N. *et al.* TILL-D: An *Aegilops tauschii* TILLING resource for wheat improvement. *Front. Plant Sci.* **871**, (2018).

7 Yu, G., Hatta, A., Periyannan, S., Lagudah, E. & Wulff, B. B. H. Isolation of wheat genomic DNA for gene mapping and cloning. in *Methods in Molecular Biology* vol. 1659 (2017).

8 Watson, A. *et al.* Speed breeding is a powerful tool to accelerate crop research and breeding. *Nat. Plants* **4**, 23–29 (2018).

9 Luo, M. C. *et al.* Genome sequence of the progenitor of the wheat D genome *Aegilops tauschii*. *Nature* **551**, 498–502 (2017).

10 Paten, B. *et al.* Cactus: Algorithms for genome multiple sequence alignment. *Genome Res.* **21**, 1512–1528 (2011).

11 Kurtz, S., Narechania, A., Stein, J. C. & Ware, D. A new method to compute K-mer frequencies and its application to annotate large repetitive plant genomes. *BMC Genomics* **9**, 517 (2008).

12 König, S., Romoth, L. W., Gerischer, L. & Stanke, M. Simultaneous gene finding in multiple genomes. *Bioinformatics* **32**, 3388–3395 (2016).

13 Shumate, A. & Salzberg, S. Liftoff: an accurate gene annotation mapping tool. *bioRxiv* 2020.06.24.169680 (2020) doi:10.1101/2020.06.24.169680.

14 Seppey, M., Manni, M. & Zdobnov, E. M. BUSCO: Assessing genome assembly and annotation completeness. in *Methods in Molecular Biology* vol. 1962 227–245 (Humana Press Inc., 2019).

15 Walkowiak, S. *et al.* Multiple wheat genomes reveal global variation in modern breeding. *Nat. 2020* 1–7 (2020) doi:10.1038/s41586-020-2961-x.

16. Li, H. A statistical framework for SNP calling, mutation discovery, association mapping and population genetical parameter estimation from sequencing data. *Bioinformatics* **27**, 2987–2993 (2011).

17. Malik, R., Brown-Guedira, G. L., Smith, C. M., Harvey, T. L. & Gill, B. S. Genetic mapping of wheat curl mite resistance genes *Cmc3* and *Cmc4* in common wheat. *Crop Sci.* **43**, 644–650 (2003).

18. Cox, T. S. *et al.* Registration of KS96WGRC40 hard red winter wheat germplasm resistant to wheat curl mite, Stagnospora leaf blotch, and Septoria leaf blotch. *Crop Sci.* **39**, 597–597 (1999).

19. Dhakal, S. *et al.* Mapping and KASP marker development for wheat curl mite resistance in “TAM 112” wheat using linkage and association analysis. *Mol. Breed.* **38**, 119 (2018).

20. Rudd, J. C. *et al.* ‘TAM 112’ wheat, resistant to greenbug and wheat curl mite and adapted to the dryland production system in the Southern High Plains. *J. Plant Regist.* **8**, 291–297 (2014).

21. Rudd, J. C. *et al.* ‘TAM 204’ wheat, adapted to grazing, grain, and graze-out production systems in the Southern High Plains. *J. Plant Regist.* **13**, 377–382 (2019).

22. Marchler-Bauer, A. *et al.* CDD: A Conserved Domain Database for the functional annotation of proteins. *Nucleic Acids Res.* **39**, D225 (2011).

23. IWGSC. Shifting the limits in wheat research and breeding using a fully annotated reference genome. *Science (80-.).* **361**, (2018).

24. Bhullar, N. K., Street, K., Mackay, M., Yahiaoui, N. & Keller, B. Unlocking wheat genetic resources for the molecular identification of previously undescribed functional alleles at the *Pm3* resistance locus. *Proc. Natl. Acad. Sci. U. S. A.* **106**, 9519–9524 (2009).

25. Holzberg, S., Brosio, P., Gross, C. & Pogue, G. P. Barley stripe mosaic virus-induced gene silencing in a monocot plant. *Plant J.* **30**, 315–327 (2002).

26. Loutre, C. *et al.* Two different CC-NBS-LRR genes are required for *Lr10*-mediated leaf rust resistance in tetraploid and hexaploid wheat. *Plant J.* **60**, 1043–1054 (2009).

27. Martin, E. C. *et al.* LRRpredictor—a new LRR motif detection method for irregular motifs of plant NLR proteins using an ensemble of classifiers. *Genes (Basel).* **11**, (2020).

28. Hayta, S. *et al.* An efficient and reproducible Agrobacterium-mediated transformation method for hexaploid wheat (Triticum aestivum L.). *Plant Methods* **15**, (2019).

29. Werner, S., Engler, C., Weber, E., Gruetzner, R. & Marillonnet, S. Fast track assembly of multigene constructs using Golden Gate cloning and the MoClo system. *Bioeng. Bugs* **3**, 38–43 (2012).

30. Lazo, G. R., Stein, P. A. & Ludwig, R. A. A DNA transformation–competent *Arabidopsis* genomic library in *Agrobacterium*. *Bio/Technology* **9**, 963–967 (1991).

31. Tingay, S. *et al.* Agrobacterium tumefaciens-mediated barley transformation. *Plant J.* **11**, 1369–1376 (1997).

32. Lewis, C. M. *et al.* Potential for re-emergence of wheat stem rust in the United Kingdom. *Commun. Biol.* **1**, (2018).

33. Bartlett, J. G., Alves, S. C., Smedley, M., Snape, J. W. & Harwood, W. A. High-throughput *Agrobacterium*-mediated barley transformation. *Plant Methods* **4**, 22 (2008).

34. Kangara, N. *et al.* Mutagenesis of *Puccinia graminis* f. sp. *tritici* and selection of gain-of-virulence mutants. *Front. Plant Sci.* **11**, (2020).

35. Stakman, EC, Stewart, DM, Loegering, W. *Identification of physiologic races of Puccinia graminis var. tritici*. *USDA ARS* (1962).

36. McIntosh, RA, Wellings, CR, Park, R. *Wheat Rusts: An Atlas of Resistance Genes*. (CSIRO Publishing, 1995).

37. Jones, H. *et al.* Strategy for exploiting exotic germplasm using genetic, morphological, and environmental diversity: The *Aegilops tauschii* Coss. example. *Theor. Appl. Genet.* **126**, 1793–1808 (2013).

38. Allen, A. M. *et al.* Transcript-specific, single-nucleotide polymorphism discovery and linkage analysis in hexaploid bread wheat (*Triticum aestivum* L.). *Plant Biotechnol. J.* **9**, 1086–1099 (2011).

39. Allen, A. M. *et al.* Discovery and development of exome-based, co-dominant single nucleotide polymorphism markers in hexaploid wheat (*Triticum aestivum* L.). *Plant*

Biotechnol. J. **11**, 279–295 (2013).

40. ZADOKS, J. C., CHANG, T. T. & KONZAK, C. F. A decimal code for the growth stages of cereals. *Weed Res.* **14**, 415–421 (1974).
