# Supplementary material for: Population genomic analysis of Aegilops tauschii identifies targets for bread wheat improvement
Source: Nat Biotechnol. 2021 Nov 1;40(3):422–31. doi: 10.1038/s41587-021-01058-4 (PMC8926922; doi:10.1038/s41587-021-01058-4)
Supplement: Supplementary file 2 — Reporting Summary [file 41587_2021_1058_MOESM2_ESM.pdf]

nature research

Corresponding author(s): Brande Wulff
Jesse Poland
Beat Keller
Alison Bentley

Last updated by author(s): Jul 27, 2021

Reporting Summary

Nature Research wishes to improve the reproducibility of the work that we publish. This form provides structure for consistency and transparency in reporting. For further information on Nature Research policies, see our Editorial Policies and the Editorial Policy Checklist.

Statistics

For all statistical analyses, confirm that the following items are present in the figure legend, table legend, main text, or Methods section.

n/a	Confirmed	
☐	☒	The exact sample size (*n*) for each experimental group/condition, given as a discrete number and unit of measurement
☐	☒	A statement on whether measurements were taken from distinct samples or whether the same sample was measured repeatedly
☐	☒	The statistical test(s) used AND whether they are one- or two-sided<br>*Only common tests should be described solely by name; describe more complex techniques in the Methods section.*
☒	☐	A description of all covariates tested
☐	☒	A description of any assumptions or corrections, such as tests of normality and adjustment for multiple comparisons
☐	☒	A full description of the statistical parameters including central tendency (e.g. means) or other basic estimates (e.g. regression coefficient) AND variation (e.g. standard deviation) or associated estimates of uncertainty (e.g. confidence intervals)
☒	☐	For null hypothesis testing, the test statistic (e.g. *F*, *t*, *r*) with confidence intervals, effect sizes, degrees of freedom and *P* value noted<br>*Give P values as exact values whenever suitable.*
☐	☒	For Bayesian analysis, information on the choice of priors and Markov chain Monte Carlo settings
☒	☐	For hierarchical and complex designs, identification of the appropriate level for tests and full reporting of outcomes
☒	☐	Estimates of effect sizes (e.g. Cohen's *d*, Pearson's *r*), indicating how they were calculated

Our web collection on statistics for biologists contains articles on many of the points above.

Software and code

Policy information about availability of computer code

Data collection

Where commercial, open source and custom code has been used to collect phenotypic data, this has been indicated in the manuscript.
Custom code has been made available from https://github.com/wheatgenetics/owwc.
Aegilops tauschii metadata was obtained primarily from: www.genesys-pgr.org

Data analysis

Bespoke code for the study is available from https://github.com/wheatgenetics/owwc
Softwares and databased used in the study during data analysis are:

Phenotyping:
ImageJ
R 3.5.1 (R Core team 2020)

Genome assembly:
MEGAHIT v1.1.3
Trimmomatic v0.238
TRITEX (Monat et al. 2019: Genome Biol. 20, 284)
CANU (Koren et al. 2017: Genome Res. 27, 722–736)
Pilon (Walker et al. 2014: PLoS One 9).

Gene annotation:
HISAT2 (default parameters)
Cactus (Version 1.0)
Tallymer subtools from the Genome Tools package (Version 1.6.1)
Augustus comparative annotation pipeline (Version 3.3.3)

BLASTp (2.3.0+)
PTREP (http://botserv2.uzh.ch/kelldata/trep-db/index.html (Release 19))
UniPoa/UniMag/UniProt: https://www.uniprot.org (Release 2016_07, downloaded: 3 Aug 2016)
AHRD pipeline (https://github.com/groupschoof/AHRD)
BUSCO (version 4.06, viridiplantae orthodb10)

SNP calling:
HISAT2 (v2.1.0)
samtools (v.1.9)
BCFtools (v1.11)

k-mer presence/absence matrix
Jellyfish (version 2.2.6 or above)

Phylogenetic tree construction
Biopython v1.77 (http://biopython.org)
iTOL (https://itol.embl.de/)

Bayesian analysis
STRUCTURE (version 2.3.4)
Structure Harvester (http://taylor0.biology.ucla.edu/structureHarvester; Web v0.6.94 July 2014, Plot vA.1 November 2012, Core vA.2 July 2014)
CLUMPAK (http://clumpak.tau.ac.il/ - beta version accessed on 11 May 2021)

FST
VCFtools (v0.1.15)

Genome anchoring
minimap2 (version 2.14 or above)

Linkage disequilibrium
PopLDdecay (v3.41)

Delimiting Cmc4 region:
Bowtie2 (v2.2.9)
BCFtools (v1.9)
GAPIT (10.1093/bioinformatics/bts444)
Ae. tauschii genome assembly (Aet v4.0; NCBI BioProject PRJNA341983)

Primer Design:
https://www.ncbi.nlm.nih.gov/tools/primer-blast/ Database: nr; Organism: Aegilops tauschii (taxid:37682)

Protein domain prediction:
CDD from NCBI: https://www.ncbi.nlm.nih.gov/Structure/cdd/wrpsb.cgi
Pfam databases (details - Sanu)
LRRpredictor (Martin et al. 2020: Genes (Basel). 11)

Gene interval size calculation:
2017 Komugi wheat gene index (https://shigen.nig.ac.jp/wheat/komugi/genes/symbolClassList.jsp)
GrainGenes (https://wheat.pw.usda.gov/GG3/)
Wheat cv. Chinese Spring assembly (IWGSC, INSDC GCA 900519105.1), EnsemblPlants

For manuscripts utilizing custom algorithms or software that are central to the research but not yet described in published literature, software must be made available to editors and reviewers. We strongly encourage code deposition in a community repository (e.g. GitHub). See the Nature Research guidelines for submitting code & software for further information.

Data

Policy information about availability of data

All manuscripts must include a data availability statement. This statement should provide the following information, where applicable:

- Accession codes, unique identifiers, or web links for publicly available datasets
- A list of figures that have associated raw data
- A description of any restrictions on data availability

The raw PacBio and Illumina sequences used for the assembly of Ae. tauschii accession TOWWC0106 have been submitted to the Genome Sequence Archive (GSA) of the National Genomics Data Center hosted by the Beijing Genomics Institute, Beijing, under the accession number CRA002681, and to NCBI under study number PRJNA730363.

The genome assemblies and annotations of TOWWC0112 and TOWWC0106 are available from the Leibniz Institute of Plant Genetics and Crop Plant Research (IPK) at https://doi.ipk-gatersleben.de/DOI/4bb6f03f-3a15-429a-b542-9962cb676e63/953a2d8a-5ade-479a-9304-6fdd12da7ce4/2/1847940088.

The 150 bp paired-end Illumina sequences for the 306 Ae. tauschii accessions, the 250 bp paired-end and mate-pair libraries for accession T0WW0112 and the RNAseq data for eight Ae. tauschii accessions is available from NCBI study number PRJNA685125.

The 150 bp paired-end Illumina sequences for the hexaploid wheat accessions and the two additional Ae. tauschii accessions used in the Cmc4 and CmcTAM112

haplotype analysis (Fig. 4; Supplementary Fig. 16) are available from NCBI study number PRJNA694980.

The k-mer matrix for 305 Ae. tauschii accessions and the tetraploid donor T. durum Hoh-501 used to generate synthetic hexaploids can be obtained from https://doi.ipk-gatersleben.de/DOI/dfc2d351-b5fe-41e6-bd6c-efe96cfcc7aa/0cef0e89-acf2-451c-8efc-a71c0368fec4/2/1847940088.

The variant call (SNP) file for 306 Ae. tauschii accessions based on the AL8/78 reference is available from Zenodo under DOI 10.5281/zenodo.4317950.

Counts of lineage-specific k-mers in wheat genome assemblies are available from Zenodo under DOI 10.5281/zenodo.4474428.

MEGAHIT assemblies for 303 Ae. tauschii accessions (including the 242 non-redundant accessions) are available from Zenodo under DOIs 10.5281/zenodo.4430803, 10.5281/zenodo.4430872 and 10.5281/zenodo.4430891.

A 29,245 bp fragment extracted from contig 00015145 of the Ae. tauschii TOWWC0106 assembly was deposited in the NCBI GenBank, along with the coordinates of the WTK4 transcript SV01, under study number MW295405.

The SrTA1662 gene and transcript sequence have been deposited in NCBI Genbank under accession number MW526949.
Figures that have associated raw data include Figs. 1-6, and Extended Data 1,2,3
Figs. 2-13 and 15-16.

Field-specific reporting

Please select the one below that is the best fit for your research. If you are not sure, read the appropriate sections before making your selection.

☒ Life sciences ☐ Behavioural & social sciences ☐ Ecological, evolutionary & environmental sciences

For a reference copy of the document with all sections, see nature.com/documents/nr-reporting-summary-flat.pdf

Life sciences study design

All studies must disclose on these points even when the disclosure is negative.

Sample size	No sample calculation was performed. We obtained as many Ae. tauschii accessions as we could get hold of and afford to sequence.
Data exclusions	We excluded genetically redundant accessions for all the analysis in the manuscript, details of which are provided in Material and Methods. GWAS was restricted to Lineage 2 of Ae. tauschii, in general. The only exception to this was GWAS for wheat curl mite.
Replication	For positive controls of stem rust resistance genes Sr45 and Sr46, published TTKSK phenotypes generated in replicates by Arora et al., 2019 (Nature Biotechnology, 37:139-143) were used; significant associations were found in the same genomic regions using both SNP GWAS and k-mer GWAS, as well as those identified by Arora et al., using AgRenSeq. For identification of stem rust resistance gene SrTA1662 we used published replicate QTHJC phenotypes (Arora et al., 2019) and also obtained new phenotypes with UK-01/TKTTF (3 to 5 replicates per genotype, depending on seed availability and germination efficiency). Both QTHJC and TKTTF identified the SrTA1662 locus. For the trichome phenotype, three replicates per genotype were used. For powdery mildew phenotypes, 3 to 4 replicates were used per genotype. For Cmc4, six replicates per genotype were used and the same genomic region was identified by both SNP and k-mer GWAS. For flowering time GWAS, three independent biological replicates were performed at different times, which all identified the same region: biological replicate 1 (Norwich, UK) included three plants per genotype, whereas biological replicates 2 and 3 (Tulln, Austria) included five plants per biological replicate. For all the experiments the attempts at replication were successful.
Randomization	Randomization was imposed for two of the flowering time experiments and for the wheat curl mite experiments.<br>For the stem rust, powdery mildew, trichome and spikelet number phenotypes, no deliberate randomization was imposed on the phenotyping procedure. The phenotypes were collected in controlled environment chambers, except for the trichome and flowering time experiments where the plants were grown in glass houses. The phenotypes for independent plants of the same genotype were generally consistent (see Table 8) and resulted in clear GWAS peaks around (i) cloned control genes (Sr45, Sr46), (ii) loci that had been previously identified by biparental genetics (e.g. SrTA1662, spikelet and trichome phenotype), (iii) around the the D-subgenome orthologue of the known flowering time regulator FLT1, and/or (iv) for which we confirmed the function of candidate genes (WTK4 and SrTA1662), thus validating our methods and conclusions.
Blinding	The persons collecting the trichome, flowering time, spikelet, rust and powdery mildew phenotypes did not have access to the genotype data. So in retrospect, the data collection was blinded.

Reporting for specific materials, systems and methods

We require information from authors about some types of materials, experimental systems and methods used in many studies. Here, indicate whether each material, system or method listed is relevant to your study. If you are not sure if a list item applies to your research, read the appropriate section before selecting a response.

Materials & experimental systems

n/a	Involved in the study
☒	☐ Antibodies
☒	☐ Eukaryotic cell lines
☒	☐ Palaeontology and archaeology
☒	☐ Animals and other organisms
☒	☐ Human research participants
☒	☐ Clinical data
☒	☐ Dual use research of concern

Methods

n/a	Involved in the study
☒	☐ ChIP-seq
☒	☐ Flow cytometry
☒	☐ MRI-based neuroimaging
